# Dynamic intracellular mechanical cues facilitate collective signaling responses

**DOI:** 10.1016/j.isci.2021.102396

**Published:** 2021-04-03

**Authors:** Bingchen Che, Wei Zhao, Yanan Liu, Dan Sun, Guangyin Jing, Jintao Bai, Xiqiao Feng, Ce Zhang

**Affiliations:** 1 State Key Laboratory of Photon-Technology in Western China Energy, Institute of Photonics and Photon-Technology, Northwest University, Xi'an 710069, China; 2 School of Physics, Northwest University, Xi'an 710069, China; 3 Institute of Biomechanics and Medical Engineering, Department of Engineering Mechanics, Tsinghua University, Beijing 100084, China

**Keywords:** Mechanobiology, Cellular Physiology, Cell Biology

## Abstract

Collective behavior emerges in diverse life machineries, e.g., the immune responses to dynamic stimulations. The essential questions that arise here are that whether and how cells *in vivo* collectively respond to stimulation frequencies higher than their intrinsic natural values, e.g., the acute inflammation conditions. In this work, we systematically studied morphological and signaling responses of population fibroblasts in an interconnected cell monolayer and uncovered that, besides the natural NF-*κ*B oscillation frequency of 1/90 min^−1^, collective signaling response emerges in the cell monolayer at 1/20 min^−1^ TNF-*α* input periodicity as well. Using a customized microfluidic device, we independently induced dynamic chemical stimulation and cytoskeleton reorganization on the stand-alone cells to exclude the effect of cell-cell communication. Our results reveal that, at this particular frequency, chemical stimulation is translated into dynamic intracellular mechanical cues through RAC1-medicated induction of dynamic cell-cell connections and cytoskeleton reorganizations, which synergize with chemical input to facilitate collective signaling responses.

## Highlights

• Dynamic intracellular mechanical cues facilitate collective cellular responses
• The dynamic chemical stimulations are translated into intracellular mechanical cues
• The synergy between dynamic mechanical and chemical signal plays crucial roles

## Graphical abstract

**Figure:**
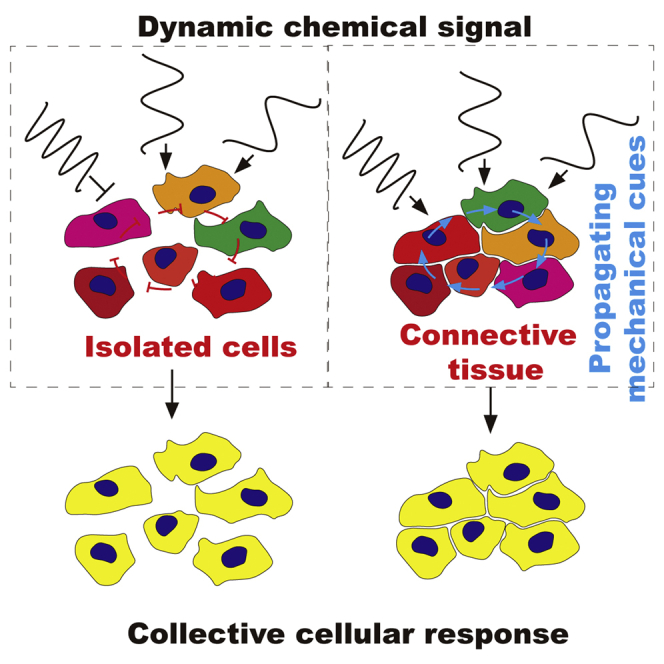

## Introduction

Collective behavior is the outcome of interactions among individual cells. The short-range cell-cell and cell-extracellular matrix (ECM) interactions allow the living tissues to undergo drastic behavioral transitions for biological functions such as migration, embryogenesis, and tumorigenesis (Roure et al., 2005; Lecaudey and Gilmour, 2006; Friedl and Wolf, 2003). The intra-cellular signaling activities are typically heterogeneous among population cells. The otherwise random signaling cascade of population cells gets coordinated by tuning the fluctuating external signals to its natural frequency (Tay et al., 2010) or through cell-cell communication to synchronize their responses (Sun et al., 2012). For example, Sun et al. reveal that collective calcium responses emerge in the crowded cell population when cells communicate via gap junctions upon ATP stimulation, leading to faster, more synchronized, and highly correlated responses as compared with the stand-alone (SA) cells.

In the crowded cellular environment of biological tissues, the local tissue architecture (Box et al., 2019) and the motion and deformation dynamics of individual cells (Pan et al., 2016) generate propagating physical signals, which affect intracellular signaling cascades. Numerous studies reveal that the physical characters of cellular micro-environment affect the long-term cellular activities associated with cell fate, including cell polarization (Copos and Mogilner, 2020; Asnacios and Hamant, 2012), division (Floris et al., 2012; Gudipaty et al., 2017), and differentiation (He et al., 2018). But the regulatory effects of mechanical cues on short-term cellular behavior, e.g., immune responses, remain unresolved. Schrader et al. reveal that abnormal ECM stiffness causes intracellular structural changes at the cytoskeleton-membrane interface and thereby blocks the transient signaling responses to targeting drugs (Frangogiannis, 2016). The fibroblast-collagen matrix can set free soluble cytokines under external mechanical actuations, which activate intra-cellular signaling pathways (Wells and Discher, 2008). These results indicate that dynamic mechanical cues resulting from inter-cell interactions can affect the intra-cellular activities. How mechanical cues facilitate population cells adapting to the ever-changing chemical environment and the correlation between collective cellular behaviors and optimal tissue functions remain unexplored.

In this work, we studied the effects of dynamic intracellular mechanical cues, which result from the collective morphological responses, on the signaling activities of population cells in an interconnected cell monolayer (ICM). By tuning the TNF-α input periodicities, we observed that fibroblasts in ICM show substantially different morphological and signaling responses as compared with the SA cells. Collective signaling activities emerge in ICM at both 1/20 and 1/90 min^−1^ TNF-*α* inputs and only at the natural frequency of NF-*κ*B signaling cascade for SA cells (i.e., 1/90 min^−1^). Meanwhile, at the particular frequency of 1/20 min^−1^, the dynamic loss and re-establishment of cell-cell connections mediated by elevated RAC1 expression generates intracellular mechanical cues, which causes collective nuclear shape changes in ICM. Using a customized microfluidic device, we reconstructed the cytoskeleton reorganization and simultaneously introduced dynamic TNF-α stimulations to the SA cells, which sophisticatedly excludes the effect of chemical information exchange. It is surprising that we found the collective behaviors within the biological tissues is a consequence of the synergy between cell-cell interactions, mechano-signaling, and NF-κB dynamics. Phase mismatching among those factors leads to disrupted collective behavior. We, therefore, conclude that the on-off process of cell-cell connections provides a feedback loop, where dynamic intracellular mechanical cues caused by TNF-*α*-induced cytoskeleton reorganization facilitate collective cellular responses to dynamic chemical stimulations. This investigation reveals a novel cascade process linking the morphological and signaling collective behaviors and provides opportunities to develop novel therapeutic strategies, utilizing controllable mechanical cues to manipulate tissue function *in vivo*.

## Results and discussion

### Morphological responses of population cells in ICM under periodic TNF-α stimulation

**Figure 1:**
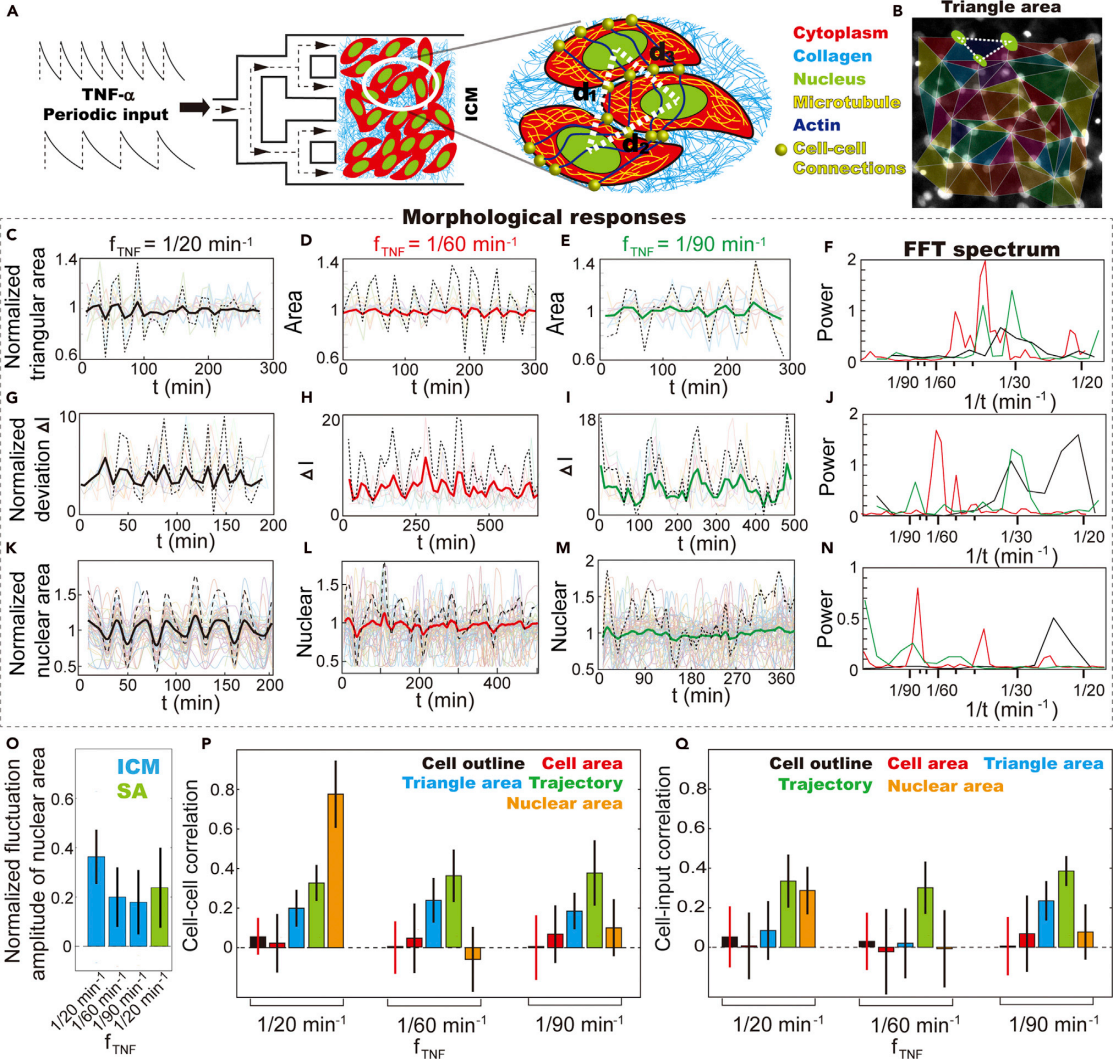
Collective morphological response of population cells in ICM upon periodic TNF-α stimulation (A) Schematic showing that the ICM was maintained in a microfluidic culture chamber and stimulated by dynamic TNF-*α* inflammatory signal of various amplitudes and frequencies. (B) Cell-cell interactions are evaluated by measuring the triangular area connecting three neighboring cells. (C–E) Relative displacement of the nucleus with respect to the neighbors causes changes in the triangular area, which reflect the cell-cell and cell-ECM interactions. The traces were normalized by their average value. The translucent lines are traces of individual cells. The solid line is the average of all traces, and the dashed line is the enlarged view of the solid line for better visualization of the fluctuation. (F) Fast Fourier transform (FFT) shows that variations in the triangular area at all TNF-*α* input periodicities share a similar dominant frequency, ranging from 1/40 to 1/30 min^−1^ (G–I) Collective vibration of nuclear centroid during cell migration within the ICM reflects deformation of the cell monolayer, which coordinates with periodic TNF-*α* stimulation. The traces were normalized by their average value. The translucent lines are traces of individual cells. The solid line is the average of all traces, and the dashed line is the enlarged view of the solid line for better visualization of the fluctuation. (J) Fast Fourier transform (FFT) shows that the dynamic ICM deformation synchronizes with TNF-*α* input. (K–M) Nuclear shape fluctuation (NSF) traces of single fibroblasts in the ICM upon stimulation. The traces were normalized by their average value. The translucent lines are traces of individual cells. The solid line is the average of all traces, and the dashed line is the enlarged view of the solid line for better visualization of the fluctuation. (N) Fast Fourier transform (FFT) shows dominant NSF frequency between 1/20 and 1/30 min^−1^, when stimulated by 1/20 min^−1^ TNF-α stimulation. (O) Fluctuation amplitude of nuclear shape changes in ICM and the stand-alone (SA) cells upon periodic TNF-α stimulation. The fluctuation amplitude of individual cells' nucleus was normalized to its time averaged area. The error bars represent standard deviation of population cell's fluctuation amplitude in nuclear area. (P) Cross-correlation analysis of the morphological responses of population cells in the ICM reveals that the collective behavior is most obvious in NSF when stimulated by 1/20 min^−1^ TNF-α input. The error bars represent standard deviation of the correlation coefficients between any 2 neighboring cells in a population. (Q) Cross-correlation analysis between the morphological responses of population cells in the ICM and TNF-α periodic stimulations reveal that contractile activities of ICM as a whole entity coordinate with TNF-α stimulation. The error bars represent standard deviation of the correlation coefficients between individual cells and the TNF-α input.

To model the crowded cellular environment in the biological tissue, we cultured high-density 3T3 fibroblasts in a shear-free microfluidic culture chamber (Figures 1A and S1). Using the integrated peristaltic pumps, nutrients and drugs loaded from the inlets can be driven to designated channels and culture chambers within tens of seconds (Zhang et al., 2019). With prolonged incubation, the fibroblasts self-organize into a compact monolayer sheet, i.e., the ICM, in which the mobility of most cells decreases dramatically (Figures S2A and S2B, Videos S1 and S2). The space between cells is filled by collagen secreted by fibroblasts (Figure S2E). The mechanical cues caused by nuclear translocation and shape transition can, therefore, be effectively transduced to the neighbors through remodeling of microtubule networks (Figures S2A–S2L and supplementary information). In the meantime, the collective movement of population cells in the ICM is regulated through the interconnective actin filaments, resulting in a correlation length of ~70 μm (Figures S2M–S2S). These results indicate that, when cell density exceeds 700 cell/mm^2^, the landscape of mechanical cues from one cell are transmitted over distances spanning multiple cell size (supplementary information). The dynamic inter- and intra-cellular mechanical cues in ICM can thus be quantified by monitoring the morphological responses of individual cells.

Video S1. Confocal fluorescence images of 3T3 fibroblasts reveal that the cell-cell interactions at low cell densities are mostly regulated by the actin filaments (as is shown in the right panel), and the microtubule networks support the cell morphology and provide volume (left panel), related to figure 1A

Video S2. Confocal fluorescence images of 3T3 fibroblasts, which were maintained in a crowded environment. It is demonstrated that the cell-cell interactions are driven by the interconnective actin filaments. Mechanical forces are transduced from one nucleus to its neighbors by inducing remodeling of microtubule networks, related to figure 1A

After the ICM was stabilized in the culture chamber, dynamic inflammatory signals of various frequencies and amplitudes were introduced to mimic the everchanging cellular environment *in vivo* (Figures 1A and S1D–S1F). In the meantime, we monitored the morphological responses of individual cells in ICM, i.e., the cell outline and surface area reflecting line strain and surface tension; the nuclear shape reflecting changes in the compressive forces caused by cytoskeleton reorganization; and the triangular area connecting three nuclear reflecting cell-cell interactions (Figures 1A, 1B, and S2T–S2V, supplementary information). Our results demonstrate that hints of collective activities emerge only in some of the morphological features. The coordinated variations of the triangle area at all TNF-*α* input frequencies leads to a correlation coefficient of ~0.2 (Figures 1B–1E) (Figure 1P). The dynamic variations suggest the emergence of synchronized mechanical cues from neighboring cells, the intracellular cytoskeleton reorganization (Harris, 2012; Khalilgharibi et al., 2019), and, to some extent, the collective movement of the interconnective actin layer (Video S3). It is intriguing that the variations in the triangle area do not coordinate with the periodic TNF-*α* stimulation (Figure 1Q). Instead, Fast Fourier transform spectrums show dominant frequencies between 1/30 and 1/40 min^−1^ regardless of TNF-*α* input periodicities (Figure 1F), suggesting that the frequency is an intrinsic character of ICM. In contrast, the morphological responses of individual cells (i.e., the cell outline and 2D projected area) are highly heterogeneous and random with dynamic TNF-*α* stimulations (Figures 1P, 1Q, S3A–S3F, and S4), reflecting mechanical cues received at the cell-cell and cell-ECM interfaces, which are timely and spatially uncoordinated among population cells. Consistently, in the time series of neighboring cells, the cross-correlation analysis reveals that the correlation coefficients of cell outline and surface area are close to zero, which are considerably smaller than that of the triangular area (Figure 1P and supplementary information).

Video S3. Collective movement of the actin filaments emerges at the initial stage of the ICM culture, and it develops into a highly interconnective layer with prolonged incubation, which coincides with the diminished mobility of individual cells in the ICM, related to figure 1A

The collective movement of population cells in ICM is disrupted by the addition of TNF-*α*. ICM of different sizes exhibit distinctive morphological responses (Figures S3G and S4). A small ICM (i.e., small quantities of cells) exhibits contractile behavior upon periodic TNF-*α* stimulation, resulting in a decrease in the overall size (Video S4). Of note, the contractile behavior starts at the marginal area (Figures S3H–S3K and Video S5). Subsequently, the dissembling of cell-ECM and cell-cell connections leads to decreased mechanical loads on the nucleus (Kim et al., 2015; Zhang et al., 2020). When the ICM is overly crowded in a confined space (i.e., >80 cells in the culture chamber of 400 *μ*m × 400 *μ*m in size), the size of the ICM remains unchanged during periodic TNF-*α* stimulation. Instead, collective oscillatory movement of nuclear and actin filaments emerges, which suggests dynamic variations in the local stress of actin networks (Figures S3I–S3K and S4). To evaluate the TNF-*α*-stimulated contractile actions of ICM, we measured the deviation of migration coordinates of individual cells from the averaged trajectory, Δ*l* (supplementary information). We observed that population cells share a similar vibrating frequency despite their distinctive long-distance migration trajectories (Figures 1G–1J and S4). That is to say that the movement of all cells is disturbed when the ICM is in contact with TNF-*α*, which results in a correlation coefficient of ~0.4 (Figures 1P and 1Q). It is plausible that TNF-*α* stimulation induces temporary disassembly in the cell-cell and cell-ECM connections (Wójciak-Stothard et al., 1998), which results in decreased local stress in the connective actin layer, and thus the contractile actions coordinating with periodic TNF-*α* stimulation. Most cells return to their original positions within 20–30 min, driven by the reorganization of actin networks. The deformation of the ICM as a whole entity can, therefore, reflect changes in the intra-cellular mechanical loads caused by the actin remodeling.

Video S4. Collective movement of fibroblasts when being maintained in confluent cell monolayer (ICM). Upon periodic TNF-α stimulation, the connective sheet partially detached from the surface, showing contractile actions, related to figures 1A and 1B

Video S5. Confocal fluorescent images and velocity field of ICM reveal that cell-ECM and cell-cell connections are disrupted at the marginal area, which are initiated by the detachment of actin filaments from the adherent surface, related to figures 1P–1Q

The intra-cellular mechanical cues can be assessed by monitoring the changes in the nuclear shape (Figures S2T–S2V) caused by the unbalanced osmotic pressure across the nuclear envelope (NE) and cytoskeleton reorganization (Kim et al., 2015). Our results demonstrate that collective behavior emerges only at 1/20 min^−1^ TNF-*α* input periodicity, showing elevated fluctuation amplitude and synchronized decrease in nuclear area (Figures 1K–1N, 1O, 1P, and S4 and Video S6) and increase in height (Figure S3M). In the meantime, H2B fluorescence intensity within the nucleus increases, suggesting more densely packed chromatin fibers. After 20 to 30 min, the nucleus restores its original shape. Of note, the nuclear shape fluctuations (NSFs) do not synchronize with TNF-*α* input, showing mode hopping between 1/20 and 1/30 min^−1^ (Figure S4A), which is considerably quicker than the nuclear deformation caused by cell-cell collision (i.e., ~2 h) (Figures S2F and S2G). The amplitude of NSF is also considerably higher at 1/20 min^−1^ than that of the SA cells and those stimulated by 1/60 and 1/90 min^−1^ TNF-*α* inputs (Figures 1O and S5A). The deviation of NSF frequency from TNF-*α* input periodicity indicates the participation of multiple cycling processes (Canolty and Knight, 2010). These results illustrate that, upon periodic TNF-*α* stimulation, the crowded cellular environment in ICM causes distinctive morphological responses of individual cells. In contrast to the SA cells, the ordered and random morphological responses of population cells and the cytoskeleton networks in ICM causes diverse propagating mechanical cues, the coupling of which leads to complex mechanical signals. Furthermore, the nuclear shape changes in ICM upon TNF-*α* stimulation is isotropic (Figure S7C), which is morphologically different from the ones caused by the remodeling of microtubule networks shown in Figure S2G. We suspect that it is the deformation of actin filaments that alters the mechanical loads on the nucleus (Figures S2T and S2V) and causes the collective NSF.

Video S6. Nuclear morphological responses of fibroblasts in the ICM when being stimulated by (from left to right) 1/20, 1/60, 1/90 min^-1^ oscillatory TNF-α input and single pulse TNF-α stimulation. Traces of single cell nuclear area (H2B-GFP) variations over time are plotted in the lower panels, related to figures 1K–M, 1O, and 1P

### Dynamic cell-cell connections cause changing mechanical loads on the nucleus

**Figure 2:**
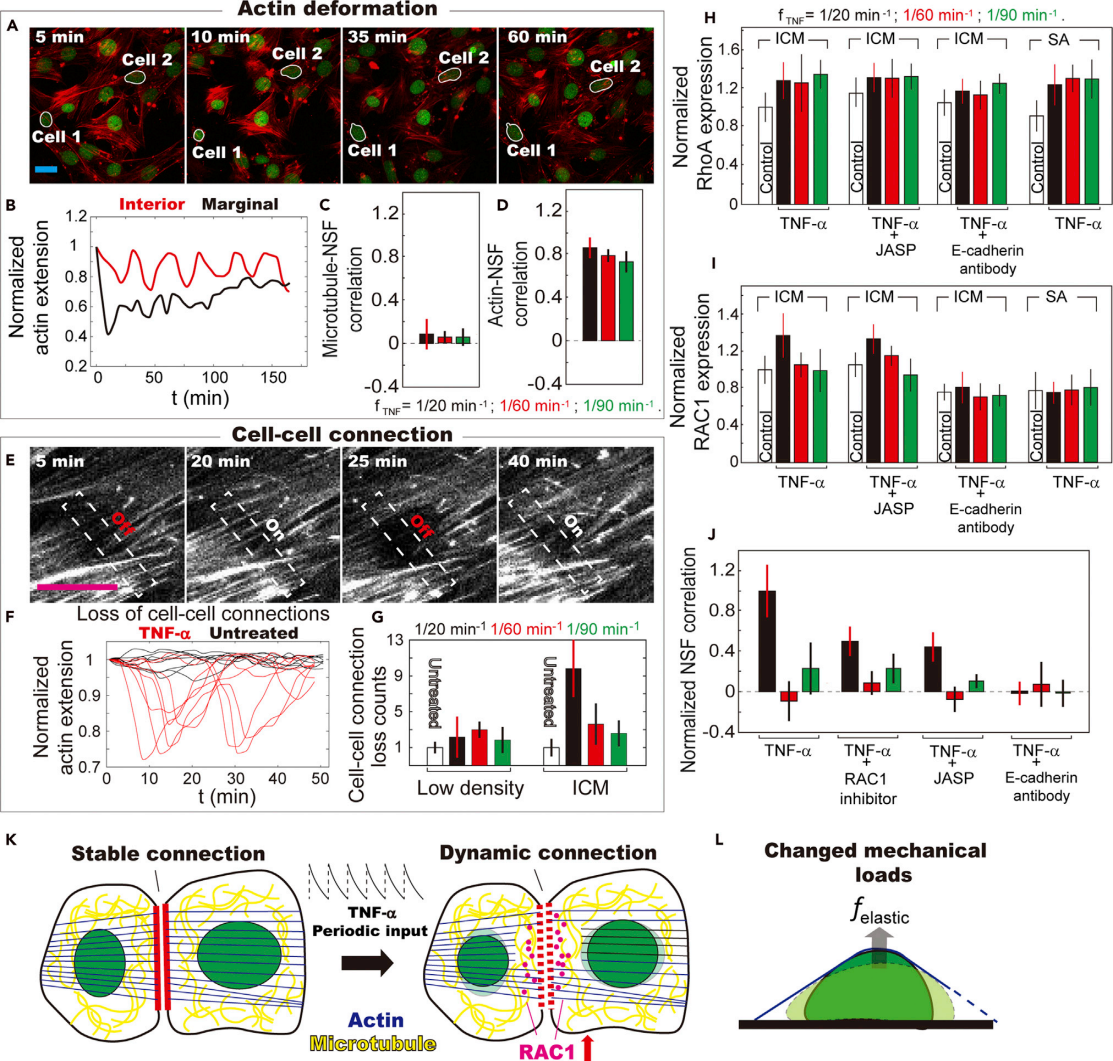
Active remodeling of cytoskeleton networks and RAC1-mediated dynamic cell-cell connections lead to collective NSF (A) Representative fluorescent images of ICM during periodic TNF-*α* stimulation show that shape transition of the nuclear (green) and actin deformation (red) are more dramatic at the marginal region. Scale bar represents 20 μm. (B) Averaged extension of actin filaments at different time points reveals that actin deformation differs at the marginal and interior area. The variations in the average actin extension were normalized by the initial value (i.e., at 0 min). (C) Cross-correlation analysis between variations in the volume of microtubule networks and NSF illustrates that remodeling of microtubule networks has trivial effects on nuclear shape. The error bars represent standard deviation of the correlation coefficients between individual cell's microtubule remodeling and NSF. (D) Cross-correlation analysis between variations in actin extension and NSF reveals that actin deformation regulates nuclear shape. The error bars represent standard deviation of the correlation coefficients between changes in individual cell's actin extension remodeling and NSF. (E) Representative fluorescent images of the actin filaments at cell-cell connections during periodic TNF-*α* stimulation show variations in the fluorescence intensity. Scale bar represents 20 μm. (F) Traces of the fluorescence intensity at the cell-cell connections demonstrate that the variations are more obvious with TNF-*α* stimulation. The variations in the average actin extension were normalized by the initial value (i.e., at 0 min). (G) Counts of the events with fluctuation amplitude of fluorescence intensity at cell-cell connections more than 10% show more frequent loss of cell-cell contacts with TNF-*α* stimulation. The error bars represent standard deviation of cell-cell connection loss counts among low density cells and in ICM. (H and I) Expression level of RhoA (H) and RAC1 (I) mRNA detected by RT-PCR and expressed as fold-change. For the data presented in (H) and (I), minimum five independent experiments were performed for each data point. The expression level of both proteins was normalized by the value of control samples, i.e., the untreated ICM. The error bars represent standard deviation of protein expression level in five independent experiments at each condition. (J) Cross-correlation analysis of the NSF of neighboring cells reveals that the collective cellular responses are disrupted by drugs regulating cell-cell connections. Correlation coefficients obtained under different conditions were normalized to the control sample, i.e., ICM treated by 1/20 min^−1^ TNF-α. The error bars represent standard deviation of the NSF correlation coefficients among population cells. (K) Schematic shows that entrainment in the Rho-associated signaling pathways leads to an elevated RAC1 expression level, which facilitates transition to more dynamic cell-cell connections. (L) Schematic shows that deformation of actin filaments leads to changing mechanical loads on the nucleus.

The cytoskeleton reorganization in response to TNF-*α* stimulation, which actively regulates the nuclear shape, is evaluated by monitoring the remodeling of microtubule networks and the deformation of actin filaments. The remodeling of individual cells' microtubule networks was assessed by reconstructing their 3D volume via confocal z stack imaging (Figure S5B). We observed that the microtubule volume of SA cells decreases down to ~80% of the initial value, when first in contact with TNF-α (Figure S5C). In contrast, no obvious change was observed in the ICM. The microtubules of SA cells regain their original volume within ~1 h (Figure S5D). The remodeling of microtubule networks during periodic TNF-*α* stimulation shows no evidence of coordination among population cells in ICM (Figures 2C, S5D and supplementary information) and does not coordinate with the nuclear shape changes (i.e., the NSF) (Figure 1C). We, therefore, conclude that the microtubule only has trivial contributions to the collective NSF.

The deformation of actin filaments upon periodic TNF-*α* stimulation was evaluated by measuring the extension of single actin filaments in individual cells, the average value of which reflects changing mechanical loads on the nucleus (Figures S5E and S5F). The addition of TNF-*α* induces drastic contractile actions of actin filaments (Figure 2A and Video S7), in contrast to the case in microtubule. Cells located at the marginal region of the ICM (e.g., cell 1) show a drastic decrease in the nuclear area as compared with the ones in the interior area (e.g., cell 2) (Figure 2A). Simultaneously, the extension of actin filaments of cell 1 decreases by ~40% (Figure 2B). The actin deformation becomes less obvious with repeated TNF-*α* stimulation, during which time cell 1 gradually regains its original volume. In contrast, cell 2, which locates in the interior region of the ICM, responds to dynamic TNF-*α* stimulation in an ordered manner. The variations in the averaged actin extension remains at a fluctuation amplitude of ~20% (Figure 2B) at 1/20 min^−1^ TNF-*α* input frequency and coordinates well with the NSF at all conditions (Figure 2D). These results suggest that actin deformation is the key regulator for the nuclear shape changes, and cells in the ICM adopt a different strategy from the SA cells in response to dynamic TNF-*α* stimulation.

Video S7. Actin deformation (red in the left panel) and dynamic cell-cell connections (right panel) shown by the fluorescent images of ICM, related to figure 2A

Shivashankar et al. discovered that TNF-*α* stimulation causes actin depolymerization (Mitra, 2017). The disappearance of the actin cap leads to decreased compressive forces on the nucleus and induces nuclear shape changes, which happens within tens of minutes. We did not observe clear fragmentation of the actin filament. Instead, by monitoring the variations of fluorescent intensity at the cell-cell connections, we found that the occurrence of dynamic loss and re-establishment of cell-cell contacts increases considerably with 1/20 min^−1^ TNF-*α* stimulation, as compared with other TNF-*α* input frequencies and the control samples (Figures 2E–2G). We then measured the gene expression level of two members of the Rho family, i.e., RhoA and RAC1, which was reported to regulate stable and dynamic cell-cell connections, respectively (Nguyen et al., 2018). Our results demonstrate that the expression level of RhoA exhibits equivalent increment by tuning TNF-*α* input frequencies (Figure 2H). In other words, 1/20 min^−1^ TNF-*α* stimulation brings no observable effect to the RhoA signaling cascade. In contrast, RAC1 expression increases only at 1/20 min^−1^ TNF-*α* input frequency (Figure 2I). The elevated expression of RAC1 at a defined TNF-*α* input frequency is similar to the entrainment in the signaling cascade (Kellogg and Tay, 2015), which is brought up by Tay, etc. to interpret coordinated and enlarged signaling activities of population cells. Therefore, we suggest that dynamic TNF-*α* stimulation activates and entrains the Rho-associated signaling pathways, leading to elevated expression of RAC1, which promotes the transition from mature to dynamic cell-cell connections (Figure 2K). As the loss of cell-cell contacts leads to changes in the extension of actin filaments and thus the mechanical loads on the nucleus (Figure 2L), the intracellular mechanical cues then cause the collective NSF shown in Figure 1K.

The hypothesis is further verified by introducing RAC1 inhibitor, Jasplakinolide (JASP), and E-cadherin antibody to the ICM. The addition of RAC1 inhibitor disrupts the collective movement of actin filaments (Figure S5G), but the collective NSF remains unaffected (Figure 2J), verifying the crucial role of RAC1. As JASP is a potent inducer of actin polymerization and known to stabilize cell-cell connections (Li et al., 2020; Holzinger, 2009), the disruption of collective NSF by JASP indicates the transition from mature to dynamic cell-cell connections as an essential linker (Figures 2J and S5G). Pretreatment of fibroblasts with E-cadherin antibody inhibits the formation of ICM and disrupts all the collective activities (Figures 2J, S5G, and S6). Possibly, when cell-cell connections are stabilized via linkers including E-cadherin, increased actin tensile stress leads to stronger compressive forces on the nucleus, which is reflected by the decreased height of the nucleus in the ICM as compared with the SA cells (Figures S5H–S5J). The complete loss or stabilization of cell-cell adhesions leads to diminished variation amplitude of the mechanical load on the nucleus, and thus the lack of changes in the nuclear shape upon periodic TNF-*α* stimulation.

### Contractility of actin filaments is essential for the collective NSF

**Figure 3:**
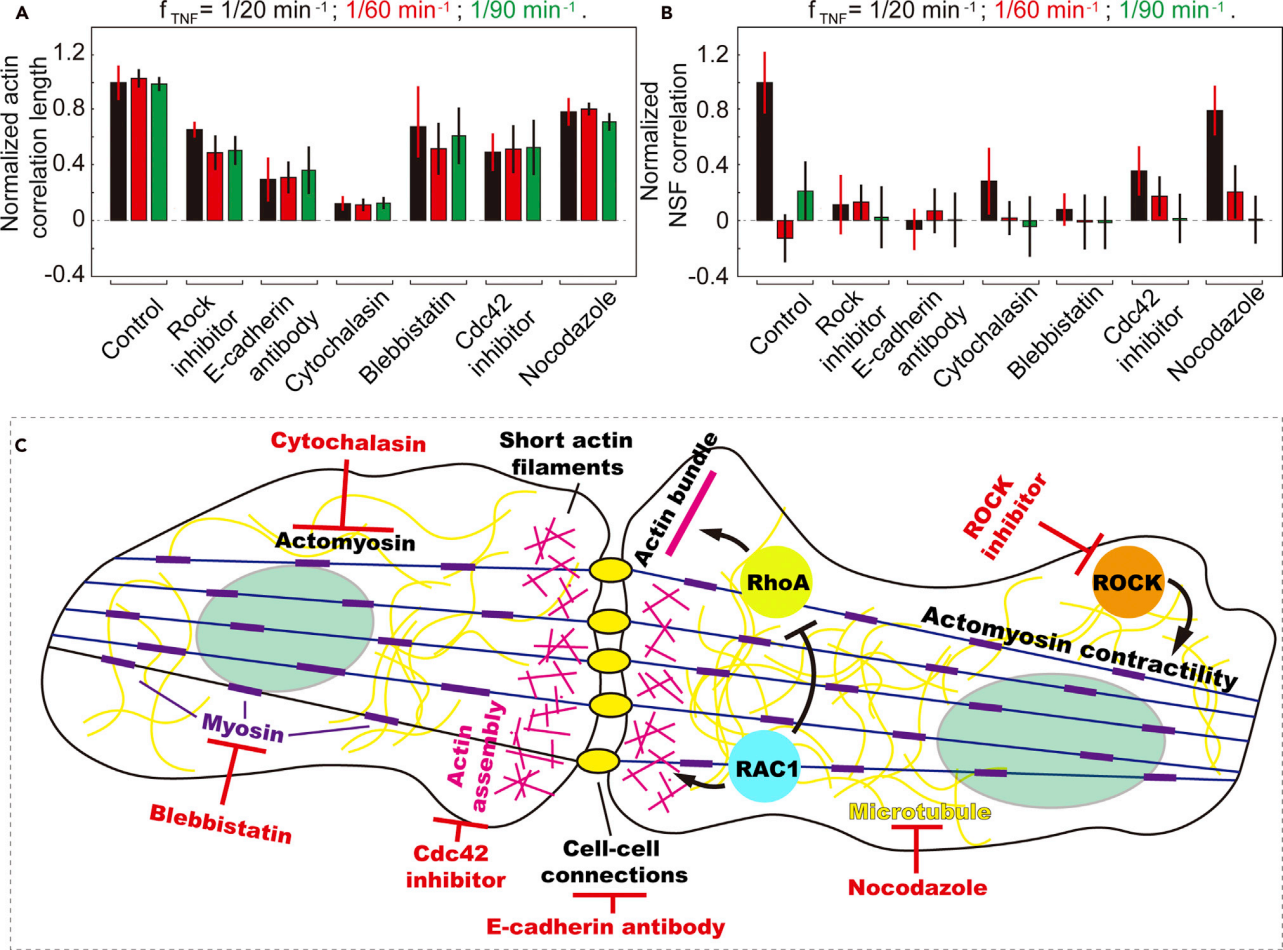
Dynamic cell-cell connections and actin contractility both play crucial roles in regulating collective morphological responses in the ICM (A) Particle image velocimetry and correlation length analysis of actin filaments in the ICM reveal that the collective movement is disrupted by drugs targeting cell-cell connections. The correlation lengths obtained under different conditions were normalized by value of the control samples, i.e., ICM treated by only 1/20 min^−1^ TNF-α. The error bars represent standard deviation of the correlation length of actin filaments in five independent experiments. (B) The collective NSF is disrupted by drugs targeting cell-cell connections and actin contractility but not the microtubule networks. The correlation coefficients obtained under different conditions were normalized by value of the control samples, i.e., ICM treated by only 1/20 min^−1^ TNF-α. The error bars represent standard deviation of the correlation coefficients of population cells' NSF in five independent experiments. (C) Schematic shows that the collective NSF is regulated by deformation of actin filaments and dynamic cell-cell connections.

As the source of the dynamically changing mechanical forces on the nucleus, the contractility of actin filaments is of crucial importance to the collective morphological responses. To identify the crucial linkers associated with actin contractility, we introduced several drugs to the ICM and monitored the morphological responses of population cells to the dynamic TNF-α stimulation, including Rho-associated protein kinase (ROCK) inhibitor Y-27632, which plays central roles in mechano-transduction (Yang, 2017) and cytoskeleton reorganization (Amano et al., 2010); cytochalasin, which is reported to depolymerize actin filaments (Casella et al., 1981); Blebbistatin, which blocks myosin II activities (Kovács et al., 2004); Cdc42 inhibitor (ML-141), which disrupts actin polymerization and assembly (Hong, 2013); and Nocodazole, which depolymerizes microtubules (Figure 3) (Xu et al., 2002). We observed that the addition of ROCK inhibitor Y-27632 disrupted the collective morphological responses in the ICM, which emerge at 1/20 min^−1^ TNF-*α* input frequency, but not the collective movement (Figures 3A and 3B). Both the collective movement and morphological responses of ICM were disrupted by cytochalasin, Cdc42 inhibitor ML-141, and Blebbistatin. In contrast, Nocodazole brings no effect to the collective activities in ICM.

Taken together, these results identify several essential factors to achieve collective NSF, i.e., the cell-cell connections and the contractility and integrity of actin filaments (Figure 3C). Conceivably, during ICM formation, stretching of actin filaments leads to increased tensile stress and forces on cell-cell connections. In the untreated ICM, the linkage between neighboring cells is stabilized enough, through the formation of long actin bundles, to maintain its integrity (Zhang, 2005; Amano, 1997), whereas with 1/20 min^−1^ TNF-*α* stimulation, one or multiple Rho-associated signaling pathways get activated and entrained, leading to elevated RAC1 expression level, which destabilizes E-cadherin-mediated cell-cell adhesion (Hage et al., 2009; Burridge and Wennerberg, 2004) and transits actin bundles to short filaments (Jiang et al., 2006). Consequently, cell-cell connections become more dynamic. The temporary loss and re-establishment of actin filaments with linkers (e.g., E-cadherin) lead to varying mechanical loads on the nucleus, and thus the collective NSF.

### Signaling responses of ICM under periodic TNF-α stimulation

**Figure 4:**
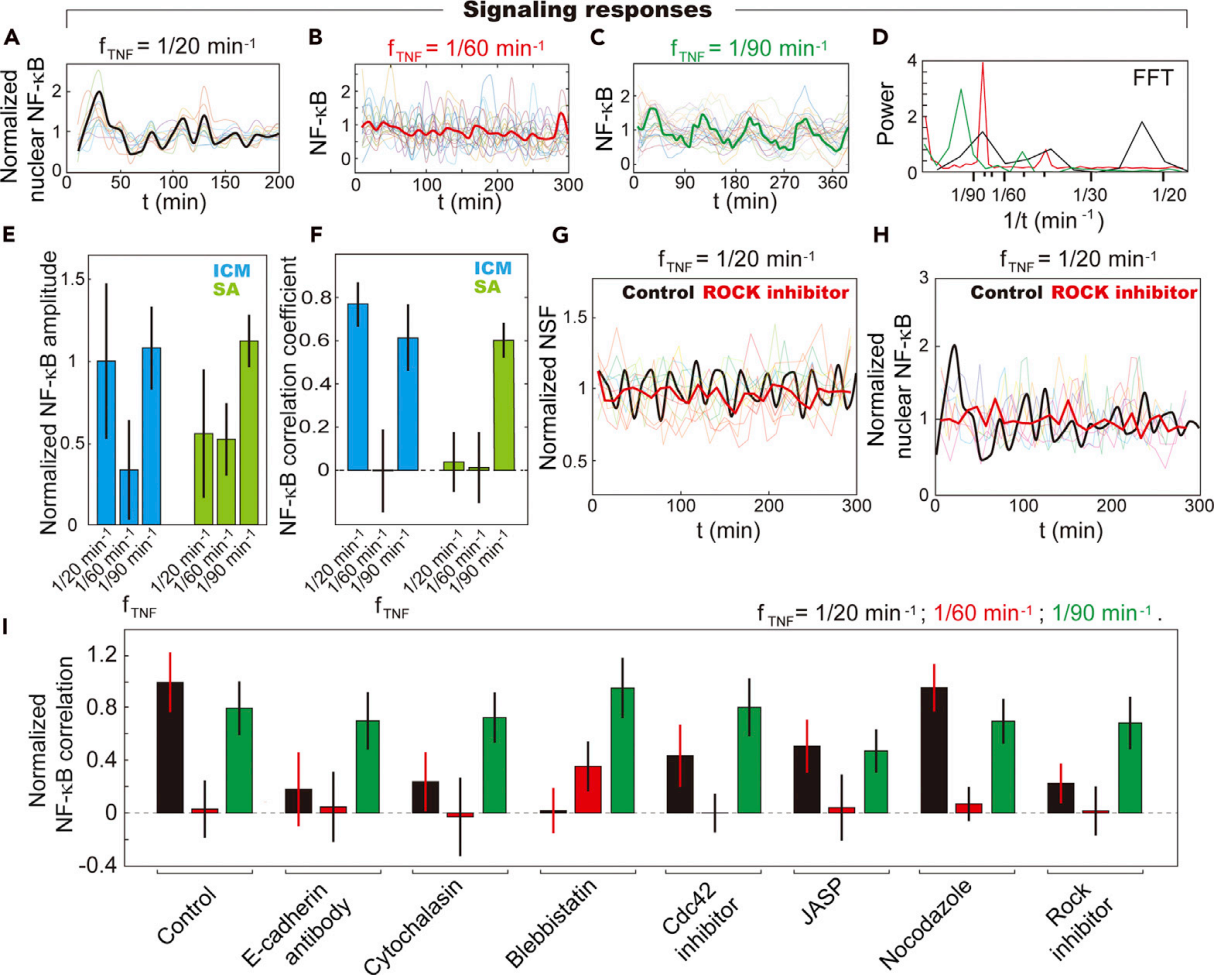
Collective signaling response of population cells in ICM upon periodic TNF-*α* stimulation (A–C) NF-*κ*B dynamics of individual cells in ICM upon periodic TNF-*α* stimulation. NF-κB traces were normalized by the average value. (D) Fast Fourier transform (FFT) shows dominant oscillation frequency close to NSF. In (A)-(C), the translucent lines are the traces of individual cells and the solid lines reflect the averaged value of single cells traces. (E) NF-*κ*B oscillation amplitude is maximized at 1/90 min^*−*1^, when getting entrained, and greatly enhanced in the ICM in response to 1/20 min^*−*1^ TNF-*α* stimulation as compared with SA cells. The NF-κB oscillation amplitude of individual cells under different conditions was normalized by value of the control samples, i.e., ICM treated by only 1/20 min^−1^ TNF-α. The error bars represent standard deviation of population cell's nuclear NF-κB fluctuation amplitude. (F) Cross-correlation analysis of the signaling responses of population cells in the ICM reveals that the collective behavior is most obvious in NF-*κ*B dynamics when stimulated by 1/20 and 1/90 min^*−*1^ TNF-*α* in ICM, and only at 1/90 min^*−*1^ TNF-*α* for SA cells. The error bars represent standard deviation of the correlation coefficients of population cells' NF-κB oscillation through NE. (G and H) NF-*κ*B and NSF traces of single fibroblasts in ICM treated with ROCK inhibitor, which is followed by 1/20 min^*−*1^ TNF-*α* stimulation. The NSF and NF-κB traces were normalized by their average values. In (G)-(H), the translucent lines are the traces of individual cells being treated by ROCK inhibitor and the solid lines reflect the averaged value of single cells traces. It is demonstrated that the application of ROCK inhibitor disrupts the collective activities of the ICM in NSF and NF-*κ*B dynamics shown in Figures 1K and 4A. (I) The collective signaling responses were disrupted by drugs, which disrupt collective NSF. The collective cellular responses at 1/90 min^*−*1^ were unaffected. The NF-κB correlation coefficient of neighboring cells under different conditions were normalized by value of the control samples, i.e., ICM treated by only 1/20 min^−1^ TNF-α. The error bars represent standard deviation of the correlation coefficients of NF-κB oscillation under different experimental conditions.

The interplay between the dynamic mechanical cues caused by cytoskeleton reorganization and TNF-*α*-activated signaling cascade is investigated by monitoring the transcription factor NF-*κ*B oscillation through the NE (Figures 4A–4D and S7A–S7C). We observed that, besides the natural frequency of NF-*κ*B signaling cascade (i.e., 90 min^−1^) (Tay et al., 2010), NF-*κ*B dynamics get entrained in the ICM at 1/20 min^−1^ TNF-*α* input, showing apparently stronger and more coordinated morphological and signaling response than the SA cells (Figures 4E–4H and S7A–S7G). Unlike other oscillators like ERK, Crz1, and NFAT4 (Albeck et al., 2013; Berridge et al., 2003; Cai et al., 2008; Dolmetsch et al., 1998), whose oscillation frequencies depend on the input signal concentration, NF-κB oscillation frequency (90–100 min peak-to-peak interval) is unchanged across a wide range of input concentrations (Longo et al., 2013; Tay et al., 2010; Turner et al., 2010). According to these results, the 1/20 min^−1^ TNF-α stimulation is sufficiently mismatched from the 90-min NF-κB natural periodicity to induce a disrupted, non-entrained NF-κB response in most cells, which is consistent with our observations on the SA cells (Figure S7A, S7D, and S7E). We, therefore, suggest that cell-cell interaction in the crowded environment of the ICM affects cellular responses, i.e., NF-κB oscillation.

In the meantime, the number of activated cells upon repeated TNF-*α* stimulation increases in the ICM as well (Figures S7H and S7I). For example, the fraction of active cells is ~20% higher in the ICM than in the SA ones when the input TNF-*α* concentration is 0.08 ng/mL. Of note, with 1/20 min^−1^ TNF-*α* stimulation, the NF-*κ*B shows an oscillating frequency of ~1/26 min^−1^, which coordinates with the collective action of NSF but deviates from the TNF-*α* input periodicity (Figures S7A and S7B). As fibroblasts in the ICM are genetically identical with those of the SA cells, these results suggest that the intracellular mechanical cues caused by cytoskeleton reorganization in the ICM affect the proceeding of biochemical signaling cascade.

The addition of ROCK inhibitor, E-cadherin antibody, cytochalasin, Blebbistatin, Cdc42 inhibitor (ML-141), and JASP, which disrupt the collective NSF at 20 min^−1^ TNF-*α* input, disrupted the collective signaling responses of population cells in the ICM as well (Figure 4I). In contrast, the entrained NF-*κ*B oscillation at the natural frequency of 1/90 min^−1^ is not affected. These results show that at 1/90 min^−1^ TNF-*α* input frequency, the mechanical cues delivered to the nucleus are mostly random and relatively small (Figures 1M, 1O, and 1P). The entrained cycling of NF-*κ*B signaling cascade is, therefore, not affected, whereas at 1/20 min^−1^ TNF-*α* input, where the intracellular mechanical forces are maximized, the collective NSF facilitates NF-*κ*B oscillation at a frequency far beyond the natural value, i.e., from 90 to 20 min^−1^.

### Stand-alone cells on chip: mechanical cues at the cell-ECM interface enhance NF-κB dynamics

**Figure 5:**
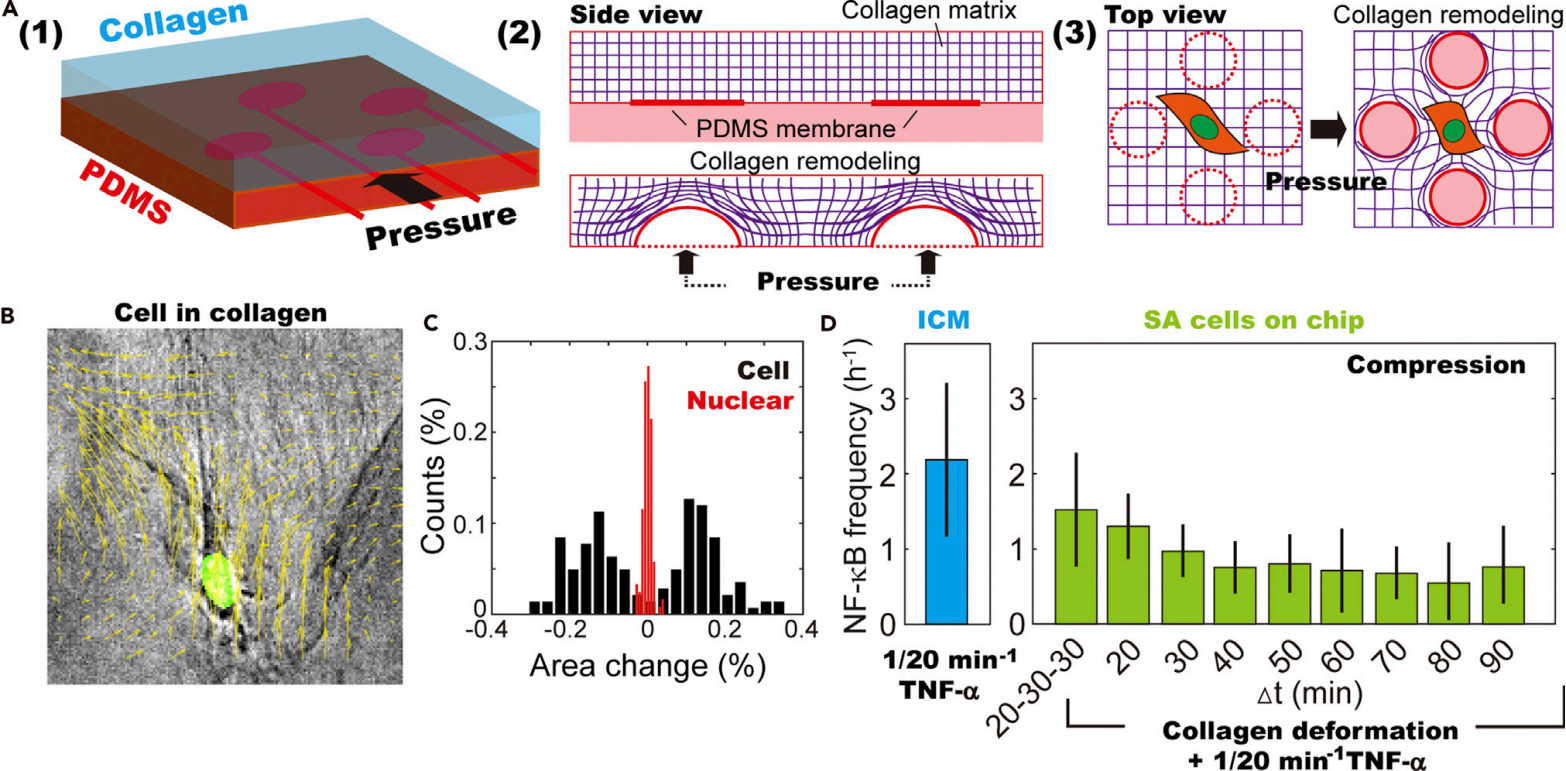
Modeling the dynamic mechanical cues at cell-ECM interfaces using a customized microfluidic device (A) Sketch of the collagen remodeling caused by repeated pressurization and relaxation of the underlying PDMS membrane, which then delivers dynamic mechanical cues to the SA cells. (B) Remodeling of the collagen matrix leads to cell shape transition (phase contrast). The nuclear shape (green) remains mostly unaffected. (C) Distribution of nuclear and cell area changes suggests that remodeling of the collagen matrix brings no obvious effects on the nuclear shape. (D) Subjected to repeated collagen remodeling at frequencies ranging from 1/20 to 1/90 min^*−*1^, 1/20 min^*−*1^ TNF-*α* is insufficient to achieve NF-*κ*B oscillation frequency comparable with ICM. NF-*κ*B dynamics is, however, enhanced, when the frequency of induced collagen remodeling is 1/20 min^*−*1^ or mode hopping between 1/20 and 1/30 min^*−*1^. The error bars represent standard deviation of population cells' NF-κB oscillation frequency through NE.

To investigate the regulatory effects of chemically induced intracellular mechanical cues, we designed a microfluidic device in which forces mimicking those in ICM can be directly applied on SA cells (Figures 5A, S8A, and S8B). In the collagen matrix, investigation on SA cells, instead of population cells, excludes the effect of cell-cell communication through chemical information exchange (Figure 5B) and uncontrollable mechanical cues caused by the movement neighboring cells (Video S8). The programmed on-off of four control channels, which are connected to the stretchable PDMS membrane underneath the collagen matrix, causes remodeling (Figures S8C–S8F and Video S9) and thus induces dynamic mechanical cues to SA cells (Figures 5B and S8G–S8I, and Video S10). The amplitude and types of induced morphological fluctuation depend on the inflating pressure as well as the location of the cells with respect to the PDMS membrane, i.e., longitudinal stretching (elongated cell morphology), lateral stretching (increased cell surface area), and compression (decreased area) (Figures S8G–S8I). Of note, the induced mechanical cues at the cell-ECM interface bring no observable changes in the nuclear shape (Figure 5C).

Video S8. When fibroblasts were maintained on the stretchable PDMS membrane, inflation of the connected control channels causes changes in the adherent surface area and consequently induces nuclear shape changes, related to figure 5A

Video S9. By pressurizing the four PDMS membranes at the bottom of the culture chamber (left and middle panel), the collagen matrix was deformed (right), which consequently changes the mechanical loads on the SA cells, related to figure 5A

Video S10. Remodeling of collagen matrix causes mechanical cues on SA cells, related to figure 5A

In the platform, TNF-*α* inputs of 1/20, 1/60, and 1/90 min^−1^ periodicities and dynamic collagen remodeling of 1/20 to 1/90 min^−1^ frequencies were then simultaneously introduced to SA cells. We observed that NF-*κ*B oscillation in responses to 1/20 min^−1^ TNF-*α* stimulation is only slightly enhanced under certain conditions, i.e., when SA cells are repeatedly compressed at 1/20 min^−1^ frequency or mode hopping between 1/20 and 1/30 min^−1^ (Figures 5D, S8J, and S8K). Even though NF-*κ*B oscillation frequency of few SA cells reaches 1/30 min^−1^, the enhanced and coordinated NF-*κ*B dynamics comparable with the ICM is not yet accomplished. The failure in reconstituting the intracellular morphological and signaling responses in the ICM suggests that the enhancement in NF-*κ*B dynamics shown in Figure 4A is closely associated with the collective NSF caused by deformation of actin filaments but not the remodeling of microtubule networks during cell-cell collision (Figure S2K).

### Stand-alone cells on chip: intracellular dynamic mechanical cues facilitate NF-κB dynamics

To verify our hypothesis that actin filaments rather than microtubules regulate signaling activities, actin deformation was induced by directly plating fibroblasts on the stretchable PDMS membrane (Figures S9A–S9G). Stretching or shrinking of cell adhering PDMS membrane surface alters cell adhesion area and consequently translocates the focal adhesions connected to actin filaments (Geiger et al., 2009; Goldyn et al., 2009), which alters the stress in actin filaments and induces NSF (Figures S9A and S9E) (Vishavkarma et al., 2014). We demonstrated that the induced NSF is morphologically similar to the individual cells in the ICM upon TNF-*α* stimulation. For example, if cells are initially plated on the inflated membrane at t = 0 min (i.e., initially), the adhesion area will rapidly decrease when the pressure is released at t = 10 min (Figure S9F). The SA cells were compressed at this moment with maximized nuclear height, and gradually restore their original conformation within ~10 min (Figures S9F and S9G). It is intriguing that we observed that the responsiveness of fibroblasts changes during induced NSF (Figures S9H–S9J). The SA cells are more responsive to TNF-*α* stimulation when being compressed, and the fraction of active cells decreases as cells go back to their original shape. Studies by Agnès, et al. revealed that the signaling cascades can be regulated by variances in cell and nuclear shape, which causes cytoskeleton reorganization and unbalances osmotic pressure across the NE(Miermont et al., 2013). Apparently, when the collective NSF emerges at 1/20 min^−1^ TNF-*α* input frequency, mechanical loads on the nucleus are repeatedly decreased, forcing population cells in the ICM to a more responsive state (Figure S9I).

**Figure 6:**
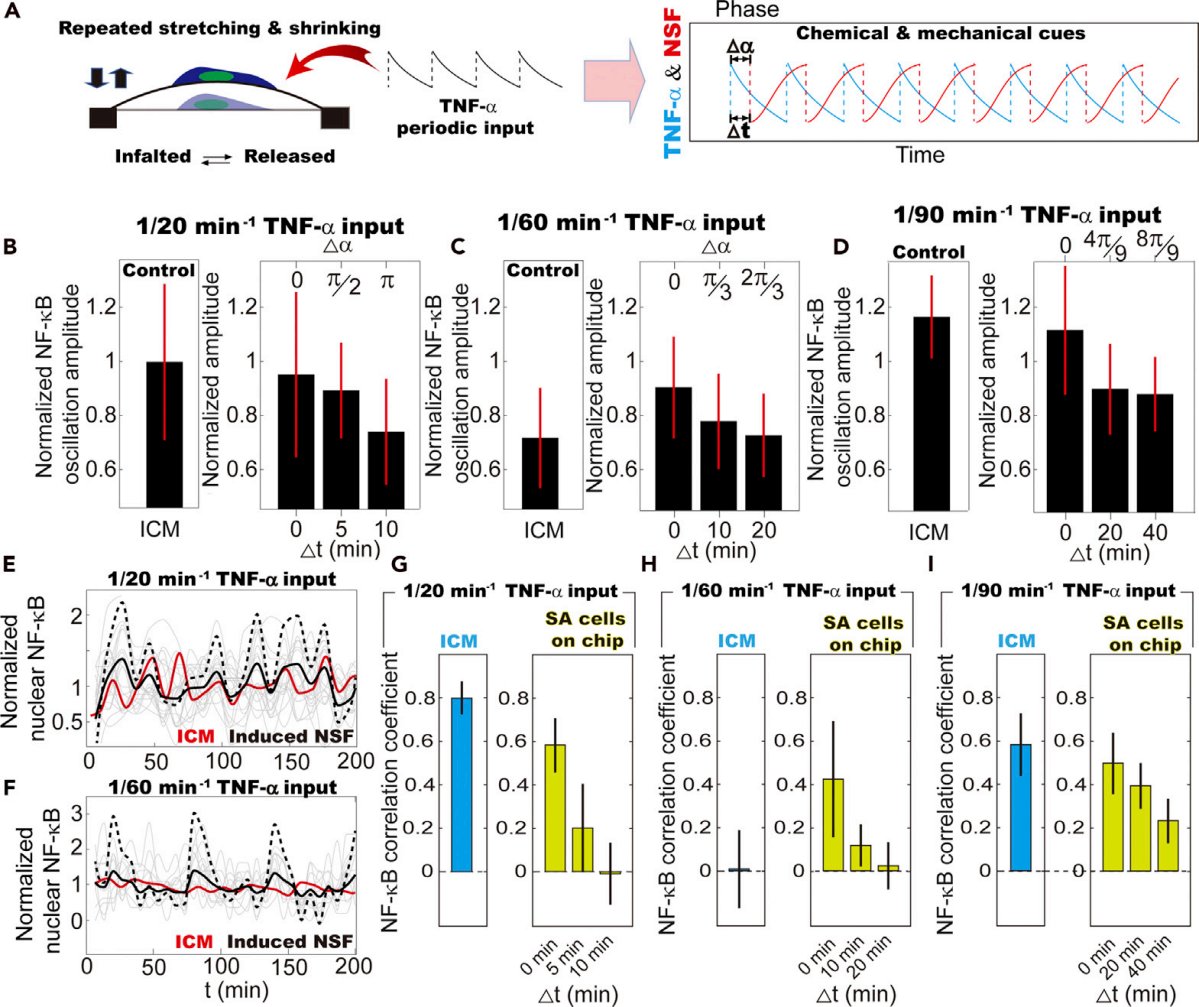
Modeling intracellular mechanical cues caused by actin deformation in the ICM: the effect of dynamic mechanical cues on NF-*κ*B oscillation of SA cells (A) NSF and TNF-*α* inputs are simultaneously introduced to SA cells using a customized microfluidic device. The time (Δt) and phase (Δ*α*) difference between minima of the nuclear area (red) and maxima of TNF-*α* concentration models the phase mismatching between mechanical and chemical cues in the ICM. (B–D) Synergy between NSF and TNF-*α* inputs induces elevated NF-*κ*B oscillation amplitude among SA cells, which is comparable with or even higher than the ones in the ICM. The NF-κB oscillation amplitudes under different conditions were normalized by value of the control samples, i.e., ICM treated by only 1/20 min^−1^ TNF-α. The error bars represent standard deviation of population cell's nuclear NF-κB fluctuation amplitude in five independent experiments. (E and F) Synergy between NSF and TNF-*α* inputs induces collective NF-*κ*B oscillation activities coordinating with TNF-*α* periodicity. NF-*κ*B dynamics averaged among all cells with induced NSF (black lines) shows higher amplitude as compared with the ones in the ICM (red lines) when being stimulated by 1/60 min^*−*1^oscillatory TNF-*α* input. The values are comparable in the ICM and on-chip in response to 1/20 min^*−*1^ oscillatory TNF-*α* input. The dashed lines are the enlarged view of the black lines. The translucent lines are the traces of individual cells with induced NSF on chip. Nuclear NF-κB traces were normalized by their average values. (G–I) Cross-correlation analysis of the signaling responses of population cells in the ICM in response to 1/20, 1/60, and 1/90 min^*−*1^ TNF-*α* input reveals that collective signaling activities emerge as long as the induced NSF coordinates with the dynamic chemical inputs. The error bars represent standard deviation of the correlation coefficients of population cells' NF-κB oscillation through NE in five independent experiments.

We then repeatedly stretched or shrunk the cell adhesion surface and simultaneously introduced periodic TNF-*α* stimulations to the SA cells (Figure 6A). During 1/20 min^−1^ periodic TNF-*α* stimulation, phase matching between the induced NSF and TNF-*α* to SA cells (Δ*t* = 0, Δ*ϕ* = 0) leads to increased NF-*κ*B oscillation amplitude comparable with the signaling responses in the ICM (Figure 6B). An enlarged phase difference (Δ*t* = 5 and 10 min; Δ *ϕ* = *π*/3 and 2*π*/3) causes decreased nuclear NF-*κ*B level. In the meantime, the otherwise random NF-*κ*B dynamics of SA cells, as shown in Figure S7E, get more coordinated with synergized NSF and TNF-*α* input, showing a shared frequency of ~1/20 min^−1^ (Figures 6E and 6G). A similar effect was observed with 1/60 and 1/90 min^−1^ TNF-*α* inputs as well (Figures 6C, 6D, 6F, 6H, and 6I). We thus conclude that the collective NSF in the ICM facilitates NF-*κ*B signaling activities, in which the phase matching between dynamic chemical inputs and NSF (i.e., the active cytoskeleton reorganization) leads to coordinated NF-*κ*B oscillation more frequently than the natural frequency of 1/90 min^−1^ (Tay et al., 2010). The entrained NF-*κ*B signaling at 1/90 min^−1^ TNF-*α* input can be disrupted by inducing phase-mismatching NSF (Figure 6I), which further emphasizes the importance of synergy between intracellular physical and chemical signals and suggests a regulatory effect of the dynamic mechanical cues.

### The feedback loop translating periodic chemical input into dynamic mechanical cues

**Figure 7:**
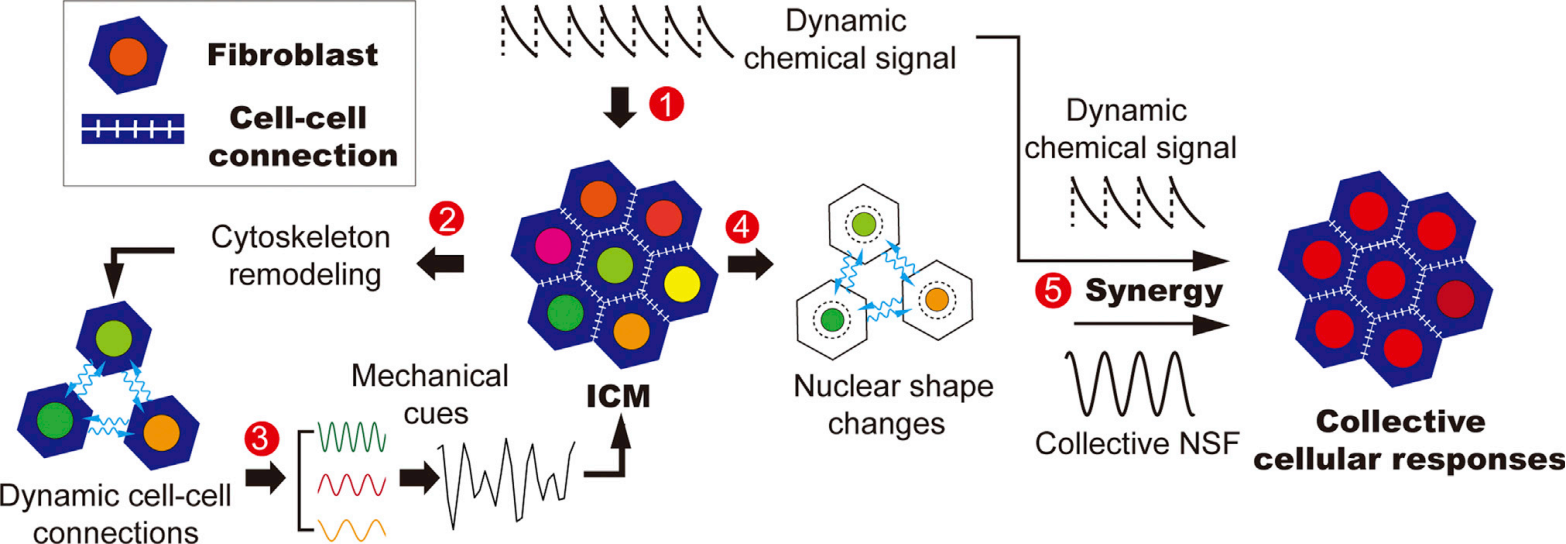
Schematic shows the feedback loop, in which dynamic intracellular mechanical cues caused by active cytoskeleton reorganization synergize with the chemical signal to facilitate collective cellular responses In the feedback loop, dynamic chemical signals are converted into mechanical cues via RAC1-mediated transition from mature into dynamic cell-cell connections.

Taken together, our results suggest a feedback loop intrinsic to the crowded cellular environment in the ICM, where dynamic mechanical cues caused by cytoskeleton remodeling facilitate collective cellular responses (Figure 7). In stages ① and ②, when the periodic chemical stimulation meets the natural frequency of signaling pathways associated with cytoskeleton reorganization, the elevated expression level of regulators (e.g., RAC1) leads to changes in the cell-cell connections from stable to a more dynamic state (Jiang et al., 2006). The loss of connections diminishes the extension of actin filaments and thus alters the mechanical loads on individual nuclei. The cyclic process continues when cells in the ICM reconnect with their neighbors (stage ③). The multi-frequency NSF (i.e., mode hopping between 1/20 and 1/30 min^−1^) may be caused by the participation of other dynamic signals, e.g., the TNF-α-caused contractile action of ICM and proceeding of mechano-signaling pathways, all of which cause changing mechanical forces on the nucleus (stage ④). As the consequence of dynamic mechanical cues feeding back to the ICM, the collective NSF synergizes with the dynamic chemical input, leading to the collective cellular responses at a frequency far beyond the natural value of the signaling cascade (stage ⑤).

## Conclusions

In this study, we quantified the collective morphological and signaling responses of population cells in an ICM, which models the crowded cellular environments in the biological tissue. Using a microfluidic chip and live cell imaging system, dynamic TNF-*α* stimulations at input frequencies of 1/20, 1/60, and 1/90 min^−1^ were introduced to either SA cells or the ICM. The correlation between the intracellular mechanical loads and collective signaling responses was investigated by simultaneously monitoring the cytoskeleton reorganization and NF-*κ*B oscillation dynamics in response to periodic TNF-*α* stimulations.

We found that, for the SA cells, NF-*κ*B oscillation gets coordinated with its amplitude reaching maximum under only 1/90 min^−1^ TNF-*α* stimulation, which is consistent with the reported natural frequency of NF-*κ*B signaling cascade, whereas in the crowded environment of the ICM, NF-*κ*B oscillation gets maximum coordination at both 1/90 and 1/20 min^−1^ of TNF-*α* input frequencies. In the meantime, at the particular TNF-*α* input frequency of 1/20 min^−1^, the RAC1 expression level is unexpectedly elevated. This leads to enhanced transition from mature to dynamic cell-cell connections and more frequent on-off of cell-cell contacts, which cause a decrease in the overall extension of actin filaments in the cell. Subsequently, the properties (i.e., characteristic frequency and amplitude) of intracellular mechanical cues are altered, leading to an amplified morphological and signaling response of the ICM to 1/20 min^−1^ TNF-*α* stimulation. These results indicate an intracellular mechanism facilitating the collective signaling responses of population cells in the ICM, in which chemical stimulations are translated into dynamic mechanical cues through RAC1-mediated active cytoskeleton reorganization.

The intracellular mechanism determining the collective morphological and signaling responses was further studied by isolating SA cells in a customized microfluidic device, which can induce remodeling of cytoskeleton networks and independently generate dynamic chemical signals. Excluding the effect of cell-cell communication, we demonstrate that, regardless of the input frequencies of mechanical and chemical stimulations, responses of SA cells on par with the ICM can be accomplished as long as the induced dynamic cytoskeleton remodeling phase matches with the TNF-*α* stimulation periodicities. This supports our finding that NF-*κ*B dynamics in ICM is only enhanced at 1/20 min^−1^ stimulation periodicity, where NSF reflecting the intracellular mechanical cues and the TNF-α input frequency are most coordinated. The emergence of collective morphological and signaling responses at a defined TNF-α input frequency of 1/20 min^−1^ implies that there exists a feedback loop in the ICM, in which the periodic TNF-*α* stimulation leads to entrainment in the RAC1-associated signaling pathways and cytoskeleton reorganization. Subsequently, the changing mechanical loads on the nucleus cause the collective NSF, which synergizes with the dynamic TNF-*α* stimulation and facilitates collective cellular responses.

Our findings reveal a novel strategy that allows the biological tissue to generate phase-locked responses to acute inflammatory signaling far beyond its natural frequency. Beside its biological significance, the regulatory effects of dynamic mechanical cues on collective cellular responses suggest new therapeutic strategies, in which dynamic mechanical signals can be induced by acoustic or magnetic field to minimize the cell-to-cell variability *in vivo* and get a coherent downstream response. Furthermore, our studies demonstrated the capacities of the presented microfluidic system in generating phase mismatching between the dynamic chemical and physical signals at milliseconds accuracy, which allows us to systematically investigate the importance of synergy between various environmental cues in regulating collective cellular responses.

### Limitations of this study

Regarding the dynamic mechanical cues, which regulate intracellular signaling activities, the limitation of this study is that we have not yet quantified these forces. One of the remaining questions is how nucleus as the mechanosensor distinguishes diverse mechanical cues. For example, nuclear deformation was often observed during cell-cell collision. Would the transduced mechanical forces affect cellular responses?

### Resource availability

#### Lead contact

Further information and requests for resources and reagents should be directed to and will be fulfilled by the lead contact, Ce Zhang (zhangce.univ@gmail.com). ***Materials availability*** This study did not generate new unique reagents.

#### Data and code availability

All relevant data are available from the authors upon request.

## Methods

All methods can be found in the accompanying transparent methods supplemental file.

## Supplemental information

Document S1. Transparent methods and figures S1–S9

## Footnotes

Supplemental information can be found online at https://doi.org/10.1016/j.isci.2021.102396.
