# Supplementary material for: Dynamic intracellular mechanical cues facilitate collective signaling responses
Source: iScience. 2021 Apr 3;24(5):102396. doi: 10.1016/j.isci.2021.102396 (PMC8091894; doi:10.1016/j.isci.2021.102396)
Supplement: Document S1. Transparent methods and figures S1–S9 [file mmc1.pdf]

iScience, Volume *24*

Supplemental information

Dynamic intracellular mechanical cues

facilitate collective signaling responses

Bingchen Che, Wei Zhao, Yanan Liu, Dan Sun, Guangyin Jing, Jintao Bai, Xiqiao Feng, and Ce Zhang

Transparent Methods

1.1 Design and Fabrication of Microfluidic Chips

We designed and fabricated the microfluidic devices for generation of dynamic chemical and mechanical cues following the standard protocol, which was reported elsewhere (*1*). Briefly, we designed our microfluidic device shown in Fig. S1, which contains 200 culture units for the maintenance and stimulation of the interconnected cell monolayer (ICM) and stand-alone (SA) cells, using AutoCAD (Autodesk Inc., San Rafael, CA, USA). Templates for control, flow and middle membrane layers are fabricated using standard UV-lithography, using SU-8 3025, SU-8 3075 (Microchem, Westborough, MA, USA) and AZ-50X (AZ Electronic Materials, Luxembourg) photoresists. To fabricate the chip, 72g of PDMS (10:1 of monomer:catalyst ratio) was mixed, de-bubbled and poured over the Trimethylchlorosilane treated patterned silicon wafer. The PDMS was then cured for 60 min at 80 ℃. Following plasma and alignment between flow, control and membrane layer, inlet holes were then punched after two-hour thermal bonding. The chip was bonded to a PDMS coated coverslip and cured for at least 24 hours at 80 ℃ before use.

The fluidic chip, which generate dynamic mechanical cues, was design using SolidWorks (Dassault Systèmes SolidWorks Corp., CA, USA). The templates for different layers were fabricated using 3D printing (CR-3040, CREALITY, China), which were smoothed using Polysher. The PDMS chip was then produced using standard soft lithography protocol as described above.

1.2 Chip Setup, Operation and Control

The glass slide carrying the microfluidic chip was cleaned and taped on a slide holder. Control channels were connected to miniature pneumatic solenoid valves (Festo, Switzerland) that were controlled with a custom MATLAB (MathWorks, US) through graphical user interface *(1)*. Optimal closing pressures of push-up PDMS membrane valves were determined individually for each chip, typically ranging from 25 to 30 psi for the chips produced using UV lithography and 5 to 10 psi for the 3D-printed ones. The cell culture chambers were treated with fibronectin (0.25 mg/mL; Millipore, Austria) for 3T3 fibroblast culture. The remaining coating solution was flushed off from the chip using either PBS or cell culture media. Cell culture media is pre-warmed on chip for overnight before cell loading.

1.3 Cell Culture and Loading

NIH 3T3 p65-/- cells were transfected with p65-dsRed and H2B-GFP for tracking and analysis of NF-κB activation and nuclear shape changes. These cells are cultured according to the established protocols (*2*). The cytoskeleton elements (i.e., actin and microtubule) were visualized by staining cells with SiR-actin kit and SiR-tubulin kit (Cytoskeleton, Inc., US). For studies on the stand-alone (SA) cells, 3T3 fibroblasts are harvested at 80% confluence with trypsin, re-suspended and loaded into chips through semi-automated loading program at cell density from 10^4 to 10^6 per milliliter depending on the desired cell density. To construct an interconnected cell monolayer (ICM), cells were cultured at ~100% confluency for 24 h either in 96-well plate before being transferred to the microfluidic device through connected tubing, or directly on chip before stimulation.

The environmental conditions are maintained using temperature control and incubator system (OKOLab, NA, Italy) to strictly 37 ℃ and >98% humidity and 5% CO_2 during the experiment, and the PDMS chip is covered with a stage top incubator connected to a humidifier and a gas exchanger.

1.4 Maintaining cells in the collagen matrix on chip

To produce collagen matrix on chip, 225 μL collagen solution (Millipore Sigma) at 3 mg/mL concentration was mixed with 45 μL 7.5% $NaHCO_3$ in MEM right before adding into the 100 μL cell solution. In order to maintain 3T3 fibroblasts in the collagen matrix at the single cell level, cells were suspended at 10^4/mL density. The collagen and cell solutions were quickly mixed right before being loaded into the fluidic device, which was pretreated to 37 ℃. The collagen solidifies and traps single cells within 5 to 10 min.

1.5 Live-cell Fluorescence Microscopy and Data analysis

For image acquisition, Nikon Ti2-ECLIPSE microscope with an automated translational stage and a digital CMOS camera (ORCA-Flash 4.0, Hamamatsu, Japan), and Nikon A1 confocal microscope were used. The stage and image acquisition were controlled via the NIS Elements software (Nikon, Japan). Bright field and fluorescence images were acquired and analyzed using a customized MATLAB program. The algorithm extracts 2D morphological features, including outline of individual cells, surface area, nuclear area, nuclear height, actin extension, and 3D microtubule volume by 3D reconstructing the confocal z-stack images. Trajectories of the individual cells were retrieved by tracking the position of nuclear centroids, using which the distance and area between neighboring cells were quantified.

1.6 Isolation of mRNA and quantification via RT-PCR

To estimate the expression level of proteins associated with cell-cell connections, 3T3 fibroblasts were firstly maintained on chip at 100% confluency for 24 h to form confluent cell monolayer, or at ~ 30% for the stand-alone cells. After repeated TNF-α stimulation for 4 h, cells were collected from the fluidic chips and fully ground in 1 ml Takara RNAiso Plus Kit (Trizol) for quantification of RAC1 and RhoA mRNA level. The supernatant after centrifuging at 12,000 rpm at 4 ℃ for 5 min was extracted twice by chloroform. The sedimentation after centrifuging at 12,000 rpm at 4 ℃ for 15 min in isopropyl alcohol was resuspended in 75% alcohol. Total RNA quantification was assayed by a nanodrop spectrophotometer. Multiple chips may be used for one test. For mRNA reverse transcription, 1 μg of RNA was used to synthesize cDNA using RT Primer Mix and Prime Script RT Enzyme Mix I (Takara). The primer sequences including RAC1, forward 5'-GCGTTGCCATTGAACTCACC-3' and reverse 5'-GAGCTGCTACGCTCACTCCATTAC-3'; RhoA, forward: 5'-AGCCTGTGGAAAGACATGCTT-3'; reverse primer: 5'-TCAAACACTGTGGGCACATAC-3'; β-actin forward (5' to 3')：TGCTGTCCCTGTATGCCTCTGG, and β-actin reverse (5' to 3')：ACCGCTCGTTGCCAATAGTGATG were used for the reverse transcriptase of mRNA target. Genes of RhoA and RAC1 were normalized to genes β-actin for mRNA analyses.

1.7 PIV analysis and the correlation analysis

The PIVLab toolbox in Matlab is used to run the PIV analysis on the video stack of ICM and the stand-alone (SA) cells. In short, the velocity correlation is calculated by

$$C_{ij} = \left\langle \frac{\overline{V_i(\vec{r})V_j(\vec{r}+\vec{R})} - \overline{V_i(\vec{r})}^2}{\overline{V_i^2(\vec{r})}} \right\rangle$$

as function of the distance $R = |\vec{R}|$, the indices *i* and *j* represent the x- and -y direction. For each image, the correlation function is calculated as a spatial average (over-line) and a second average is taken over time (bracket) sequence of the snapshots.

1.8 Reagents and antibodies

For dynamic chemical stimulation, TNF-a input concentration is 1 ng/ml if not otherwise mentioned (Cyagen, US). For the interference of cell-cell connections, the concentrations of used drugs are 1 μM for Jasplakinolide (JASP) (Maokangbio, China); 50 μg/ml E-cadherin antibody (abcam, Shanghai); 50 μM Rac1 inhibitor NSC23766 (MilliporeSigma, US); 10 μM Rho-associated protein kinase (ROCK) inhibitor Y-27632 (abcam, Shanghai); 20 μM cytochalasin D (abcam, Shanghai); 20 μM Blebbistatin (MilliporeSigma, US); 10 μM Cdc42 inhibitor (ML-141) (MilliporeSigma, US); 10 μM Nocodazole (Beyotime, China).

References

1. M. A. Unger, H. P. Chou, T. Thorsen, A. Scherer, S. R. Quake, Monolithic microfabricated valves and pumps by multilayer soft lithography. Science. 288, 113–116 (2000).
2. Tay, S., Hughey, J.J., Lee, T.K., Lipniacki, T., Quake, S.R., and Covert, M.W. (2010). Single-cell NF-kappaB dynamics reveal digital activation and analogue information processing. Nature 466, 267-271.

2. Supplementary information

2.1 Cell-cell collision leads to the remodeling of microtubule networks and nuclear deformation

In 2013, Stephen R. Quake, etc. reported that migration of population cells is regulated by cell-cell collision, which results in pseudopod collapse and complex migratory behaviors (*1*). As cell-cell collision causes mechanical cues transduced to the nuclear through cytoskeleton networks (*2*), we expect that the intracellular activities (e.g., the chromatin reorganization) are also affected. To investigate the regulatory effects of cell-cell interactions, different numbers of 3T3 fibroblasts were maintained and monitored in real-time within the shear-free culture chambers of a microfluidic device (Fig. S1 and Fig. S2a). We observed that the migration speed of population cells is substantially lowered at high cell densities (Fig. S2b), where most individual cells are confined by the crowds (Movie S1 and Movie S2). With prolonged incubation at high cell densities >1000/mm^2 for 24 h, cell mobility further decreases as compared to the freshly plated cell population (Fig. S2b). Consistently, reconstructed 3D fluorescent images of cytoskeleton networks show that the space in between the neighboring nuclear is mostly occupied by the microtubule networks (Fig. S2h). The lattice network, where fibroblasts are embedded in, is further stabilized by continuous secretion of collagen, which ensures the structural integrity (*3*) and inhibits cell migration (Fig. S2c-e). The fibroblasts eventually self-organize into a monolayer sheet, i.e., the interconnected cell monolayer (ICM), where mechanical cues caused by nuclear migration and shape transition can be effectively transduced the neighbors. Notably, within the crowded cellular environment, changes in the relative position of one cell with respect to another (Fig. S2f), and variations in cell volume (e.g., during cell division) (Fig. S2g) lead to nuclear deformation of neighboring cells. While, in the less crowded cellular environment, cell-cell collision mostly results in cell and nuclear translocation (Fig. S2k and S2l). Estimated by overlapping nuclear outlines at different time points, we demonstrated that the occurrence of nuclear shape transitions, whose amplitude is greater than 10% of the total area, is considerably higher in ICM than the stand-alone (SA) population cells (Fig. S2i and S2j). These results demonstrate that cell-cell collision in the crowded cellular environment leads to nuclear deformation, and the transduction of mechanical forces from one nuclear to another indicates that the space in between is fully occupied. Intriguingly, the occurrence of cell-cell collision and the nuclear deformation in ICM further decreases with prolonged incubation (Fig. S2b and S2i), suggesting stronger confinement and intercellular connectivity (Fig. S2m).

2.2 Collective activities in ICM is mediated by the interconnective actin filaments

We then analyzed the velocity field in ICM using OpenPIV, an open source particle image velocimetry (PIV) software (Fig. S2n) (*4*). With the window size of ~ 60 μm by 60 μm, we observed that collective activities emerge in the displacement of nuclear at cell densities higher than 400 cells/mm^2 (Fig. S2o-p), showing correlation distance R up to nearly 70 μm. As cell-cell connection, which is mediated by cadherins and actin filaments (*5*), plays central roles in regulating collective migration of population cells (*6*), we then looked at the collective behavior of actin networks in the ICM. It was observed that at a cell density of ~ 500 cells/mm^2, actin filaments are highly interconnective and mobile at the initial stage of culture on chip (Fig. S2q-s and Movie S3). Collective behavior emerges instantaneously at 0 h, showing correlation length of ~ 60 μm (Fig. S2r). After 24-hour incubation, the mobility of actin filaments decreases dramatically (Fig. S2q and Movie S3), which is consistent with the declined cell mobility observed in the ICM shown in Fig. S2b. In the meantime, the collective behavior remains, showing correlation length of ~ 70 μm (Fig. S2s). These results suggest that the collective activities and the propagation of mechanical cues within the ICM is mediated by the collective movement and deformation of actin filaments, which changes the intra-cellular mechanical loads.

2.3 Dynamic mechanical cues reflected by morphological features of individual cells

The dynamic mechanical cues can be reflected by changes in the morphological features of individual cells (Fig. S2t-v). For example, the shape changes of the whole cell reflect changes in the extension of actin filaments (Δl), which lead to diminished stress on the nuclear (Fig. S2t), and reorganization of microtubule networks (Fig. S2u), resulting in either nuclear deformation or displacement depending on the cellular environment. The mechanical

forces from actin filaments can be simplified using $f_{actin} = \sum_{n=1}^{N_{actin}} k_{actin} \cdot \Delta l_n$, where N_{actin} is the number and k_{actin} is the linear elastic coefficient of the deformed actin filaments. As microtubules are ~300 times stiffer than actin filaments (*7*), the microtubule networks transduce mechanical cues from ECM and neighboring cells without buckling. Since the microtubule fills in volume of individual cells (Fig. S1f-h) and shows no sign of interconnection between neighboring cells, the remodeling of microtubule networks can be directly reflected by cell shape changes (Fig. S2h). In the meantime, the space in between cells is filled by collagen in ICM (Fig. S2e), and the volume between individual nucleus is mostly filled by microtubule (Fig. S2f-h). The movement and deformation of individual cell can directly affect its neighbors (Fig. S2f-g). Changes in the relative position of one cell with respect to another (Fig. S2f), and variations in cell volume (e.g., during cell division) (Fig. S2g) can therefore cause cytoskeleton remodeling and nuclear deformation of neighboring cells. We estimated the mechanical forces by measuring the total area changes between cells (Fig. S2u). For example, with unchanged d_1 and d_2, the variation in the distance between Cell 1 and Cell 3 brings changes in the area in between three cells $\Delta S_{microtubule} + \Delta S_{collagen}$ and elastic force $F_{triangle} = k_m \cdot \Delta S_{microtubule} + k_c \cdot \Delta S_{collagen}$, where k_m and k_c are the surface elastic coefficients. Notably, the mechanical forces are delivered to all cells in direct contact of the triangular area. Considering the fact that cell membrane is considerably softer than the cytoskeleton elements, the effect of dynamic variations in membrane surface tension caused by the changing cell area is neglectable.

Besides cytoskeleton remodeling, the depolymerization of actin filaments can also generate intra-cellular mechanical cue and cause nuclear deformation (Fig. S2v). Shivashankar, et al. reported that simultaneously with the biochemical signaling events (e.g., NF-κB nuclear localization), TNF-α stimulation activates mechano-signaling and causes actin depolymeri-zation (*8*), which changes the mechanical loads on the nuclear.

2.4 Propagating mechanical cues estimated by cell movement trajectories

To evaluate the TNF-α-stimulated contractile actions of ICM, the trajectory of individual cell (x_1, x_2, … x_n) and (y_1, y_2, … y_n), where n is the total frame number, is averaged every 5 to 10 steps depending on the frame rate, e.g., $x_i^{'}$ = $(x_{i+1} + x_{i+1} + \cdots x_{i+5})/5$ and $y_i^{'}$ = $(y_{i+1} + y_{i+1} + \cdots y_{i+5})/5$. The deviation of cell migration coordinates from the averaged trajectory Δl can then be calculated from its minimum distance to the piecewise cubic interpolation curve of $(x_1^{'}, x_2^{'}, \cdots x_{n/5}^{'})$ and $(y_1^{'}, y_2^{'}, \cdots y_{n/5}^{'})$ (Fig. S3l).

2.5 Calculation of correlation between the responses of neighboring cells

In this study, the correlation of signaling and morphological responses has been calculated, e.g., between the morphological responses of neighboring cells (Fig. 1p), between the periodic TNF-α and morphological response (Fig. 1q), between cytoskeleton remodeling and NSF (Fig. 2c-d), between NSF of neighboring cells (Fig. 2j). For instance, when we calculated the correlation of NF-κB nuclear localization dynamics between two neighboring cells, the cross-correlation function can be defined as:

$$correlation(\Delta t) = \overline{NF_1^{'}(t)NF_2^{'}(t+\Delta t)} / \sqrt{\overline{[NF_1^{'}(t)]^2} * \overline{[NF_2^{'}(t+\Delta t)]^2}}$$

where $NF_1(t)$ and $NF_2(t+\Delta t)$ are the nuclear NF-kB fluorescence intensity in cell 1 and 2, respectively. $NF_1^{'}(t) = NF_1(t) - \overline{NF_1(t)}$ and $NF_2^{'}(t+\Delta t) = NF_2(t+\Delta t) - \overline{NF_2(t+\Delta t)}$ are the fluctuations of NF-kB signals in cell 1 and 2, where $\bar{\cdot}$ indicates temporal averaging accordingly.

References

1. S. Vedel, S. Tay, D. M. Johnston, H. Bruus, S. R. Quake, Migration of cells in a social context. *Proceedings of the National Academy of Sciences* **110**, 129 (2013).

2. F. Alisafaei, D. S. Jokhun, G. V. Shivashankar, V. B. Shenoy, Regulation of nuclear architecture, mechanics, and nucleocytoplasmic shuttling of epigenetic factors by cell geometric constraints. *Proceedings of the National Academy of Sciences of the United States of America* **116**, 13200-13209 (2019).

3. K. Gelse, E. Pöschl, T. Aigner, Collagens--structure, function, and biosynthesis. *Advanced drug delivery reviews* **55**, 1531-1546 (2003).

4. Z. J. Taylor, R. Gurka, G. A. Kopp, A. J. I. T. o. I. Liberzon, Measurement, Long-duration time-resolved PIV to study unsteady aerodynamics. *IEEE Transactions on Instrumentation Measurement* **59**, 3262-3269 (2010).

5. M. Cavey, T. Lecuit, Molecular bases of cell-cell junctions stability and dynamics. *Cold Spring Harbor perspectives in biology* **1**, a002998 (2009).

6. J. M. Wood, M. F. Olson, Collective migration: spatial tension relief. *Current biology: CB* **22**, R125-127 (2012).

7. T. J. Mitchison, Compare and contrast actin filaments and microtubules. *Molecular biology of the cell* **3**, 1309-1315 (1992).

8. A. Mitra *et al.*, Cell geometry dictates TNFα-induced genome response. *Proceedings of the National Academy of Sciences of the United States of America* **114**, E3882-e3891 (2017).

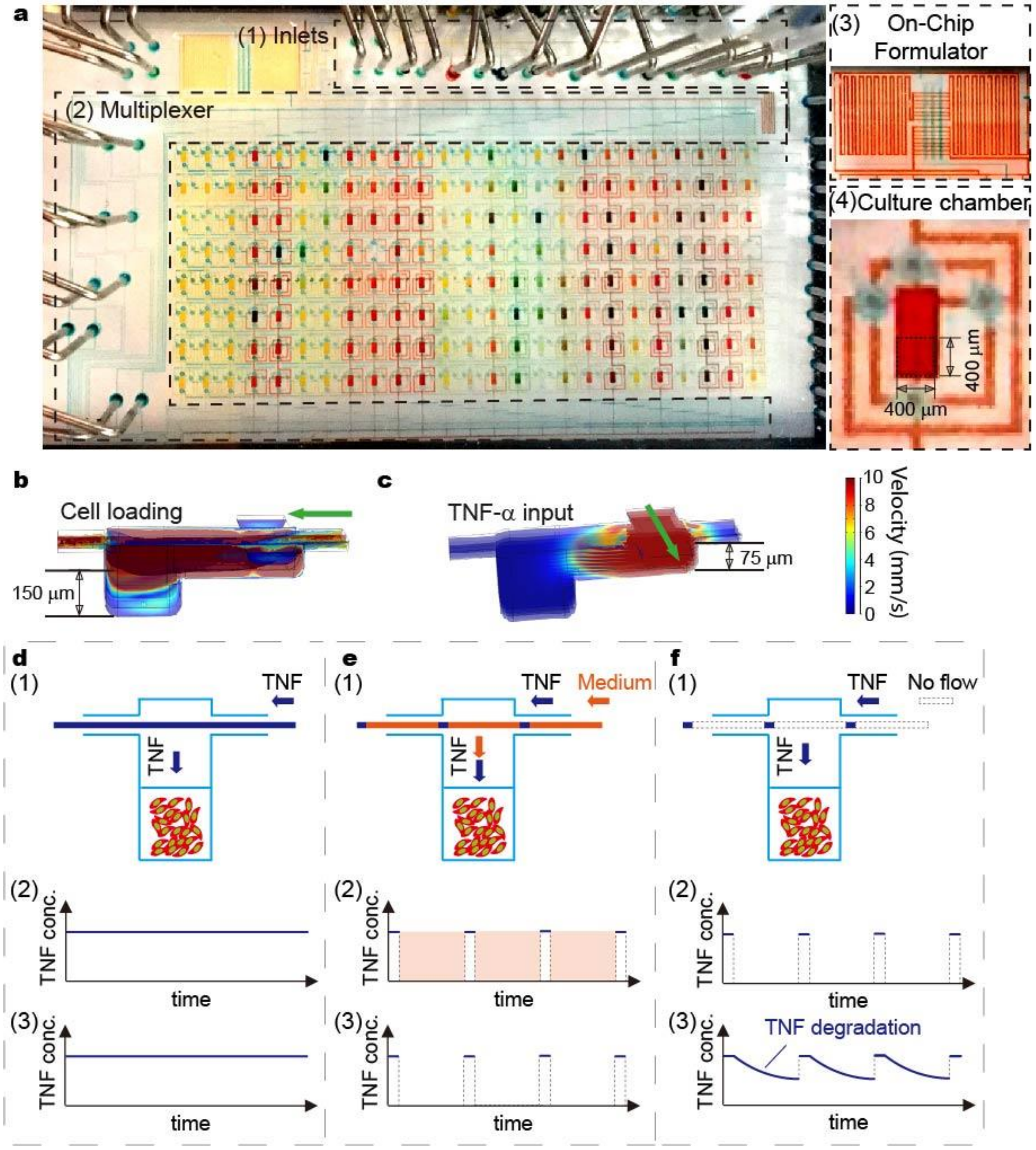

Figure S1 Fibroblasts in ICM and stand-alone (SA) cells are maintained in a shear-free microfluidic device, Related to Figure 1a. a, Automated cell culture system for dynamical live-cell analysis. (1) The microfluidic device contains 15 inlets; (2) a multiplexer, which allows control of 200 culture units; (3) an on-chip formulator with nanoliter accuracy; (4) and shear-free culture units controlled by 4 active valves (blue). The culture units are composed by 2 parts, a culture chamber of 400μm×400μm×150μm in dimension and a buffering level of 400 μm×400μm×75μm. b, Two-layer cell culture chamber allows firstly quick cell loading. c, Nutrients and TNF-α are delivered to cells in a shear-free manner. d-f: Dynamic inflammatory signals generated using microfluidic module: d, Continuous delivery of fresh TNF-α solution to cultured cells in diffusion-based mode maintain TNF-α concentration in cellular microenvironment. e, Timely replacement of TNF-α solution by culture medium allows generation of pulsatile TNF-α input. f, Programed delivery of TNF-α solution generated damped oscillatory TNF-α signal due to TNF-α degradation.

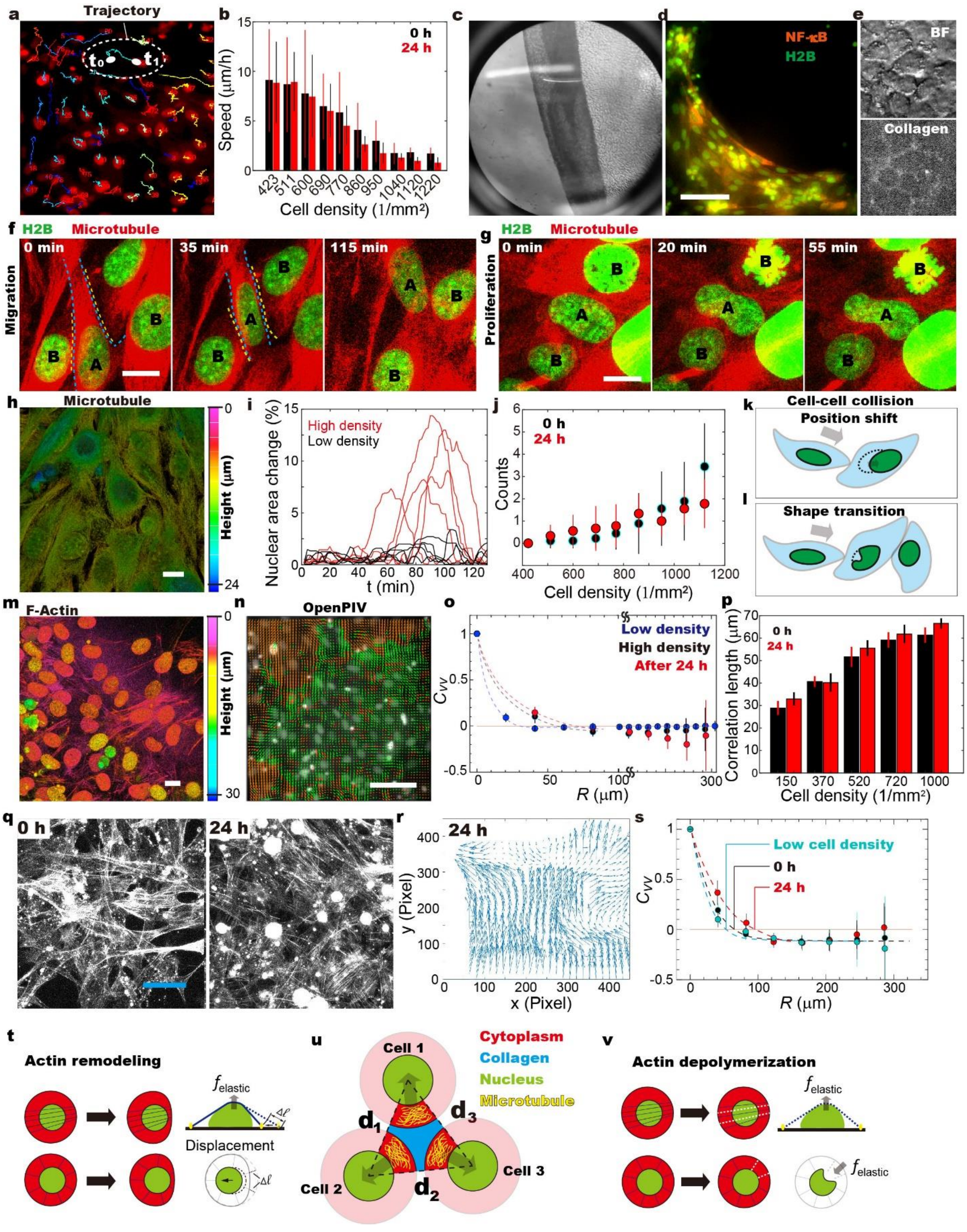
a
Trajectory
b
0 h
24 h
Speed (μm/h)
Cell density (1/mm²)
c
d
NF-κB
H2B
e
BF
Collagen
f H2B Microtubule
Migration
0 min
35 min
115 min
g H2B Microtubule
Proliferation
0 min
20 min
55 min
h Microtubule
Height (μm)
i
High density
Low density
Nuclear area change (%)
t (min)
j
0 h
24 h
Counts
Cell density (1/mm²)
k Cell-cell collision
Position shift
l
Shape transition
m F-Actin
Height (μm)
n OpenPIV
o
Low density
High density
After 24 h
R (μm)
p
0 h
24 h
Correlation length (μm)
Cell density (1/mm²)
q 0 h
24 h
r 24 h
y (Pixel)
x (Pixel)
s
Low cell density
0 h
24 h
R (μm)
t
Actin remodeling
f elastic
Displacement
u
Cell 1
Cell 2
Cell 3
Cytoplasm
Collagen
Nucleus
Microtubule
v
Actin depolymerization
f elastic

Figure S2. Dynamic mechanical cues caused by cytoskeleton reorganization in the ICM, Related to Figure 1a-b. a. Trajectories of 3T3 fibroblast nuclear centroids show distinctive mobilities of single cells in the interconnected cell monolayer (ICM). b. Mobility of individual cells were evaluated by measuring the displacement of nuclear in 1 hour. It is demonstrated that cell mobility drops substantially at high cell density, i.e., higher than 1000 cells/mm^2. There are at least 5 repeats for each cell density. c. Folded ICM layer in 96-well plate shows that fibroblasts form a connective sheet. d. ICM layer maintained in the shear-free microfluidic chip (see also Supplementary Video 1). e. Bright field (BF) and fluorescent images showing that the gaps between neighboring cells are filled by collagen fibers. f,g Representative confocal fluorescence images of nuclear deformation, (c) when cell-A passed the gate of 2 cell-B, and (d) when there is considerable volume increase in the neighboring cell-B due to division. h. Reconstructed 3D image of the nucleus and microtubule networks, where color represents height of the structure, demonstrate that the microtubule networks fill in the volume surrounding the nucleus. i. Comparison of nuclear shape transition (i.e., the absolute value of nuclear area changes) at low (dark lines) and high cell densities suggests that nuclear deformation is considerably amplified in the crowded cellular environment. j. The number of cells, which go through nuclear shape change over 10% in amplitude, shows close correlation with the cell density. k,l. Schematic shows that cell-cell collision leads to nuclear translocation of neighboring cells at low cell density (k), and nuclear shape transition at high cell density (l). m. Reconstructed 3D image of the nucleus and actin filaments demonstrate that actin locates mostly at the bottom of the ICM. n. Velocity field of nuclear movement in ICM, which was analyzed using OpenPIV software. o. Correlation between velocity vectors of nuclear indicates that mechanical cues from one cell can be transduced to other cells, which are ~ 60 μm away. p. The correlation length of nuclear velocity field linearly correlates with the cell density, which can be up to 70 μm in ICM. q. Fluorescent images of the actin filaments in ICM at different timepoints after being plated in the 96-well plate. r. Velocity field of actin filaments in ICM after 24 h. s. Correlation between velocity vectors of actin networks indicates that the collective movement in ICM is mediated by the interconnective actin filaments, which reaches ~ 60 μm at the initial stage of culture on chip. t. Schematic shows that cell shape changes lead to remodeling of actin filaments, resulting elastic forces on the nuclear. u. Cell-cell collision in the crowded environment leads to deformation in microtubule and collagen networks, which transduces mechanical forces to the nuclear. The dynamic mechanical cues can be estimated by measuring the cell-cell distance, and the triangular area in between three nuclear. v. Actin depolymerization leads to changed mechanical loads on the nuclear, and consequently causes nuclear deformation. Scale bars denote 50 μm in Fig. d; 10 μm in Fig. f, Fig. g, Fig. h and Fig. m; 100 μm in Fig. n; 40 μm in Fig. q.

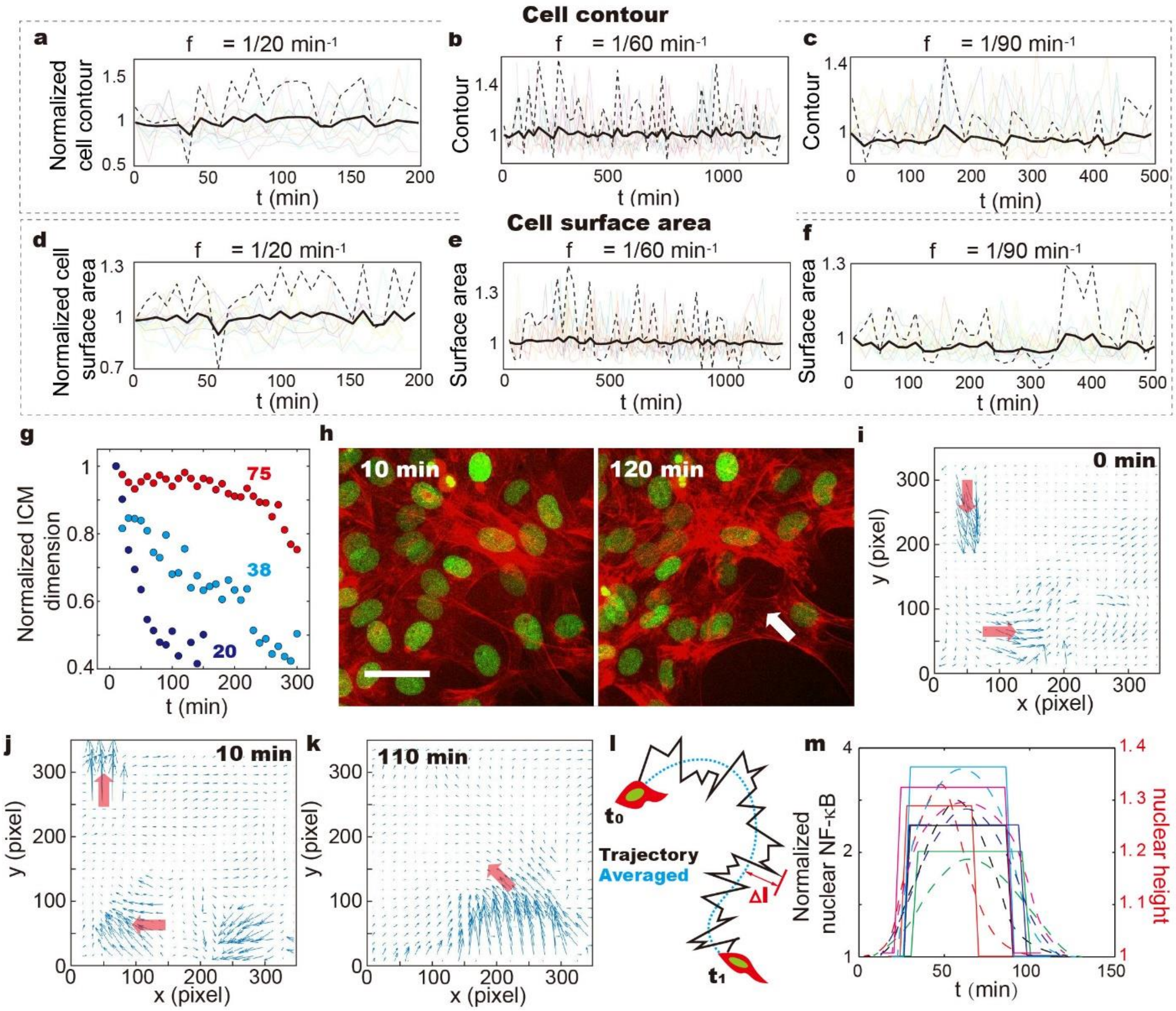

Figure S3. Morphological responses of fibroblasts in the interconnective cell monolayer (ICM), Related to Figure 1p-q. a-c. Measuring the perimeter of individual cells in ICM reveal the line strain, which shows no collective behavior. d-f. The variations in 2D area of single cell surface reflect the fluctuating surface tension. In Fig. a-f, The translucent lines are traces of individual cells. The solid lines are the average of all single cell traces, and the dashed line is the enlarged view of the solid line for better visualization of the fluctuation. g. The contractile behavior depends on the size of ICM. All values in the traces were normalized by the initial ICM dimension before TNF-α stimulation. For example, the ICM containing ~20 cells shrink down to ~40% of its original size. h. Representative confocal fluorescence images at the edge of ICM. As is indicated in Movie S5, the contractile activities are initiated by the detachment of actin filaments from cell-cell and cell-ECM connections (indicated by the arrow). i-k. Velocity field of actin filaments in ICM at the marginal area of ICM. l. ICM deformation is evaluated by measuring the distance of cell migration trajectory to the averaged curve, fluctuation of which reflect vibration of nuclear centroids. m. Variations in the nuclear height (solid lines) coordinate with the NF-κB dynamics (dashed lines). Scale bar denotes 20 μm.

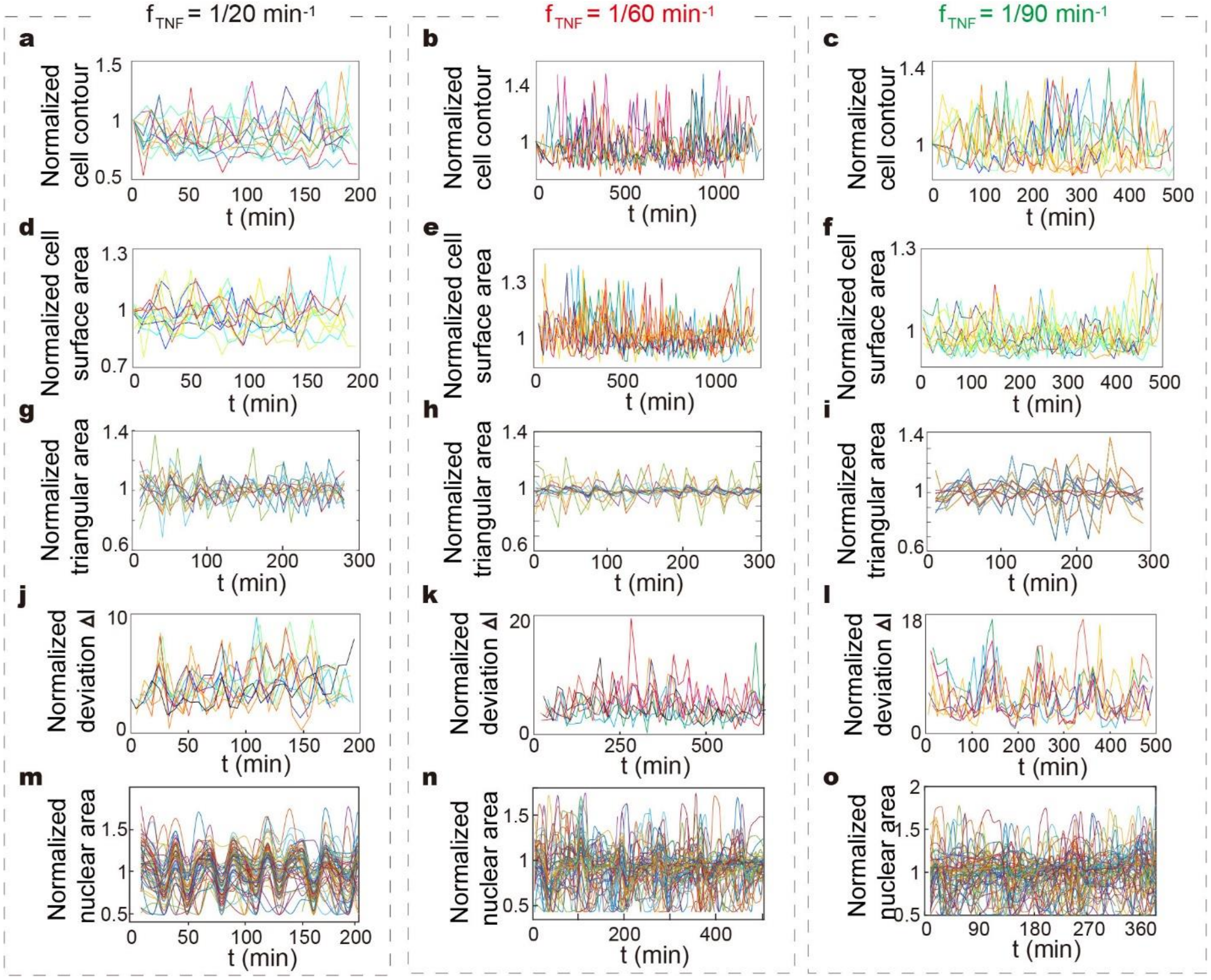

Figure S4: Morphological response of population cells in ICM upon periodic TNF-α stimulation, Related to Figure 1c-e, g-i, k-m and o-q. (a-c) Contour, (d-f) 2D surface area of individual cells in ICM changes during 1/20 min^{-1}, 1/60 min^{-1} and 1/90 min^{-1} TNF-α stimulation. (g-i) Relative displacement of nuclear with respect to the neighbors causes changes in the triangles area shown in Fig. 1a, which reflect the cell-cell and cell-ECM interactions. (j-l) Collective vibration of nuclear centroids in ICM reflects deformation of cell monolayer, which coordinates with periodic TNF-α stimulation. (m-o) Nuclear shape fluctuation (NSF) traces of single fibroblasts in ICM upon periodic TNF-a stimulation. For better visualization, all contours were normalized to initial value of individual cells, and other morphological features normalized by the average value of individual traces. Traces from individual cells are color coded.

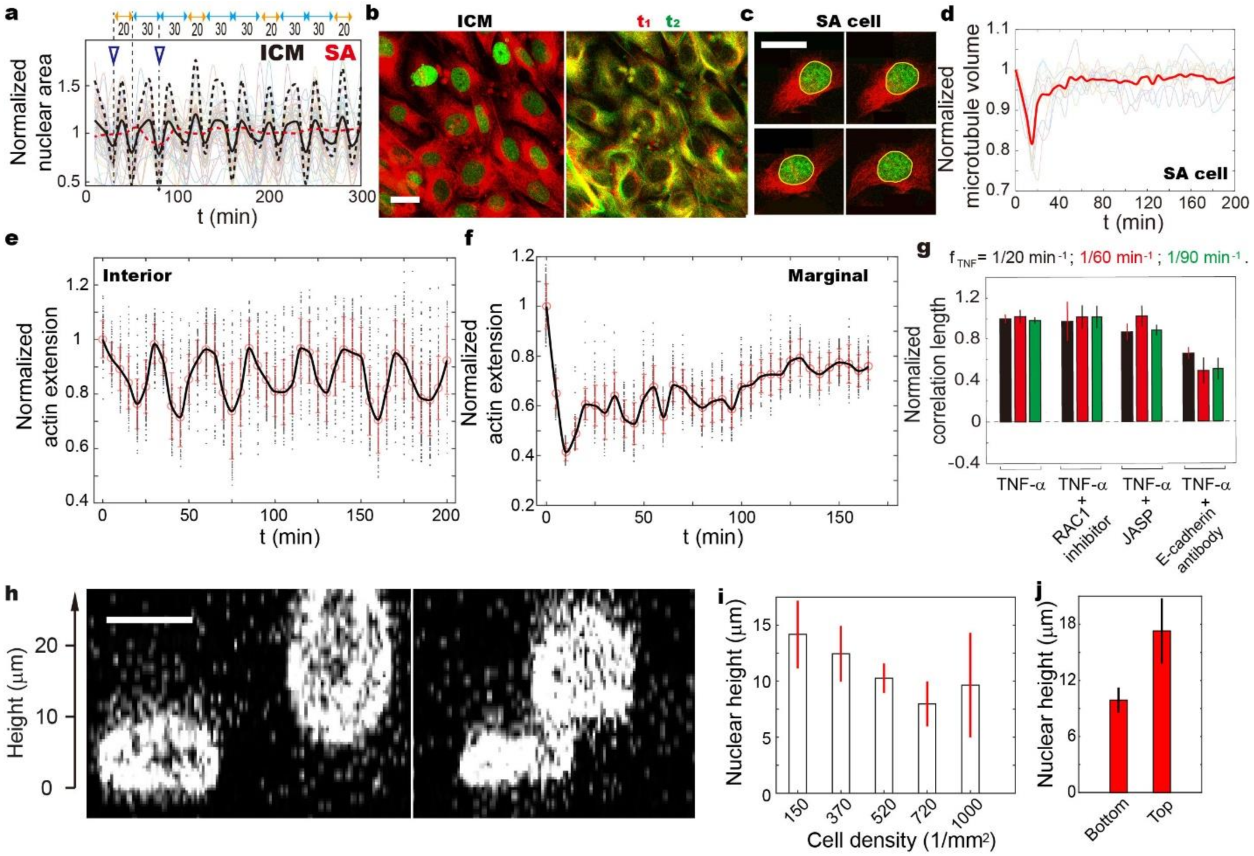

Figure S5. Cytoskeleton reorganization of fibroblasts in ICM and the SA cells upon TNF-α stimulation, Related to Figure 1o and Figure 2j. a. NSF traces of single fibroblasts in ICM upon stimulation by 1/20 min^{-1} TNF-α input. The individual NSF were normalized by the average value of each trace. The translucent lines are the traces of all participating cells, and the solid line is the averaged value of all traces. The red dashed lines represent averaged trace of stand-alone (SA) fibroblasts. b. Representative fluorescent images of the ICM, where nuclear (green) and microtubule (red) are immune-stained, demonstrate that there are no obvious volume changes in the microtubule of individual cells. c. Stand-alone (SA) cell show decrease in size when being firstly in contact with TNF-α. Elevated level of the microtubule fluorescence intensity suggest increases in the local density. d. Traces of the microtubule volume of individual cells reveal that the microtubule network remodeling of population cells in ICM are not coordinated. The traces were normalized by the initial value for better visualization. e,f. By measuring the extension of actin filaments (black dots) of individual cells in ICM and stand-alone (SA) cells, our studies reveal that deformation of actin filament leads to variations in the mechanical loads on the nuclear. In contrast to the ~20% value of the averaged actin extension, the fluctuation amplitude of SA cells is less than 10% upon periodic TNF-α stimulation, and thus considerably smaller mechanical forces on the nuclear. The traces were normalized by the initial value for better visualization. The black dots are the extension of individual actin filaments. The red circles and bars are the average value of individual cell's actin filaments and the standard deviation, respectively. The black lines are the smoothed traces of variations in actin extension. g. Correlation length of ICM shows that the collective movement of actin filaments is disrupted by E-cadherin antibody, which disrupts cell-cell connections. All values were normalized to the control value, which was obtained by treating ICM with only 1/20 min^{-1} TNF-α. h. Cross-sectional images demonstrate that cells sitting on the PDMS substrate show considerably lower height than the ones at the top of ICM. i. The height of the nuclear is inversely correlate with the cell density, suggesting the formation cell-cell connection causes compressive forces on the nuclear. j. Cells embedded in the interconnective actin networks (Bottom) show an average height of ~ 7 μm, which is considerably lower than the ones sitting on top of ICM, i.e., ~ 15 μm. Scale bars represent 20 μm.

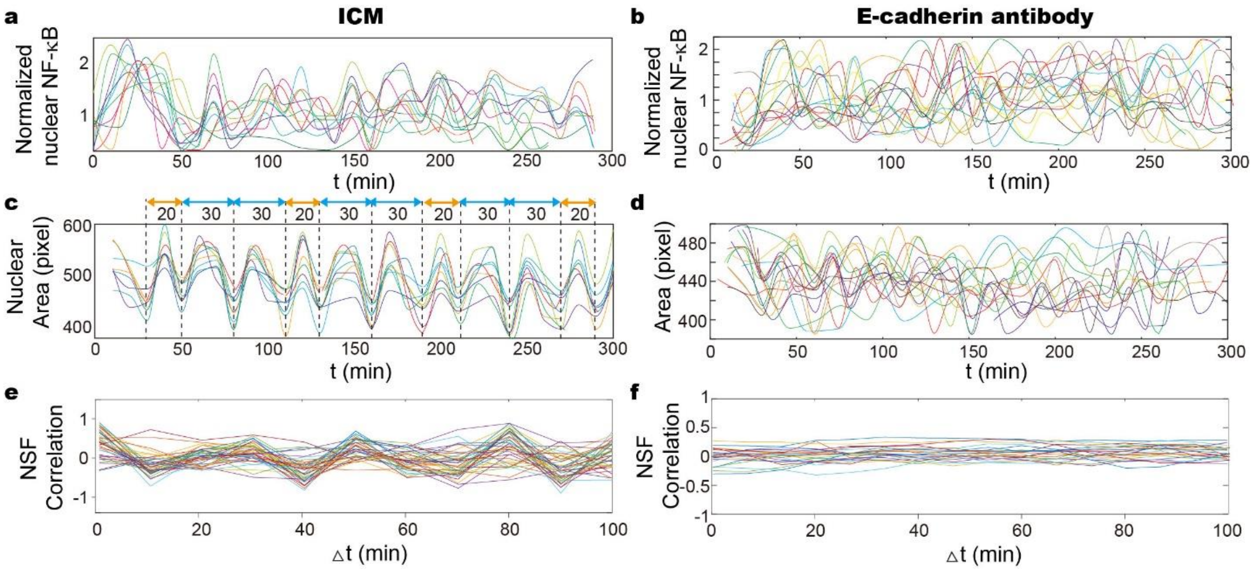

Figure S6: The collective activities in response to TNF-α stimulations were disrupted with the addition of E-cadherin antibody, Related to Figure 2j. a-d. Traces of variations in nuclear NF-κB and nuclear area show that the nuclear localization of NF-κB transcription factor is synchronized in ICM with 1/20 min^{-1} TNF-α stimulation (a,c). The synchronization as well as the formation of ICM is disrupted with the addition of E-cadherin antibody (b,d). Nuclear NF-κB traces were normalized by the average value. e,f. Cross-correlation analysis between NSF time traces of neighboring cells shows that when lag Δt=0, the NSF of individual cells positively correlate with their neighbors with 1/20 min^{-1} TNF-α stimulation (e). While, the distribution seems to center at zero with the addition of E-cadherin antibody (f), suggesting collective NSF is disrupted when cell-cell interconnections are blocked. Traces from individual cells are color coded.

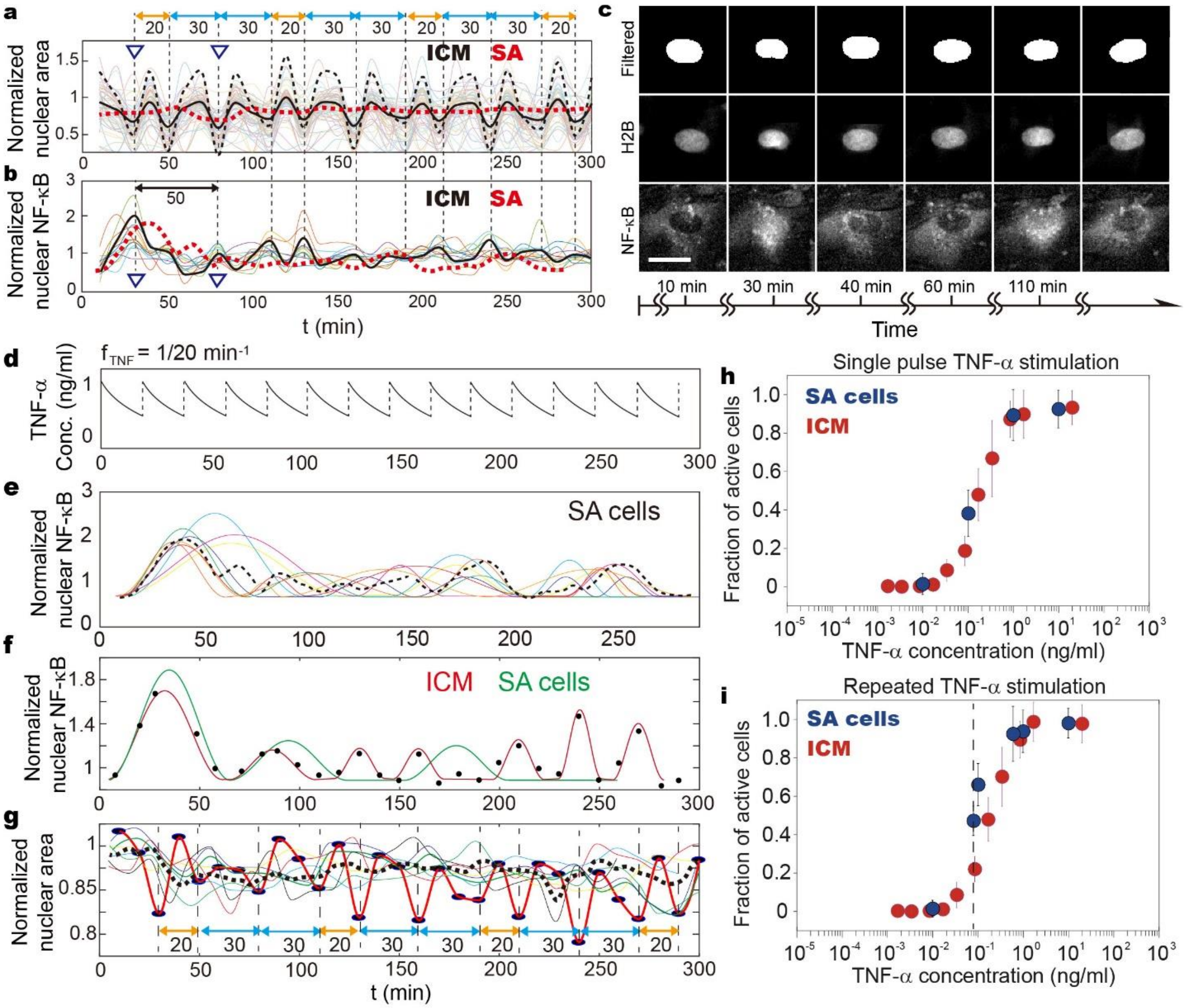

Figure S7. Responsiveness of fibroblasts in ICM and the SA cells, Related to Figure 4a-d. a,b. NF-κB and NSF traces of single fibroblasts in ICM upon stimulation by 1/20 min^{-1} TNF-α input. Solid lines reflect NF-κB dynamics and nuclear area variances averaged among all participating cells. The black dashed lines are the enlarged view of those solid lines, showing subtle variations in NSF, and the red dashed lines represent averaged NF-kB trace of stand-alone (SA) fibroblasts. c. NF-κB dynamics and NSF of single cells in ICM are monitored in real-time using fluorescence microscopy. Images show NF-κB (p65-dsRed) nuclear localization and nuclear shape changes upon periodic TNF-α stimulation. The images of H2B-GFP are processed using real-space bandpass filters to extract morphological features including perimeter and area, etc. d-g. NF-κB dynamics and NSF traces of ICM and SA cells when being exposed to 1/20 min^{-1} TNF-α stimulation. It is demonstrated collective activities only emerge in ICM, where amplitude of NSF and NF-κB oscillation is greatly elevated. In Fig. e, the traces of individual cells are presented as the color-coded solid lines, and the dashed line is the average of all traces. In Fig. f, the averaged traces of NF-kB dynamics of ICM (red) and SA cells (green) are compared. In Fig. g, the color-coded solid lines are the nuclear area traces of SA cells. The dashed line is the average of all SA cell traces. The black dots and red solid line are the averaged trace of population cells in ICM. h-i. Fraction of cells responding to single pulse, and repeated TNF-α stimulation (1/20 min^{-1} frequency). It is demonstrated that similar number of fibroblasts in ICM (blue) respond to single pulse TNF-α stimulation as the SA cells (red). While, higher number of responsive cells was observed in ICM as compared to SA cells upon stimulation by repeated TNF-α stimulation. The dashed line shows the position of 0.08 ng/mL TNF-α concentration. The NSF and NF-κB traces were normalized by their average value. Scale bar in Fig. c denotes 20 μm.

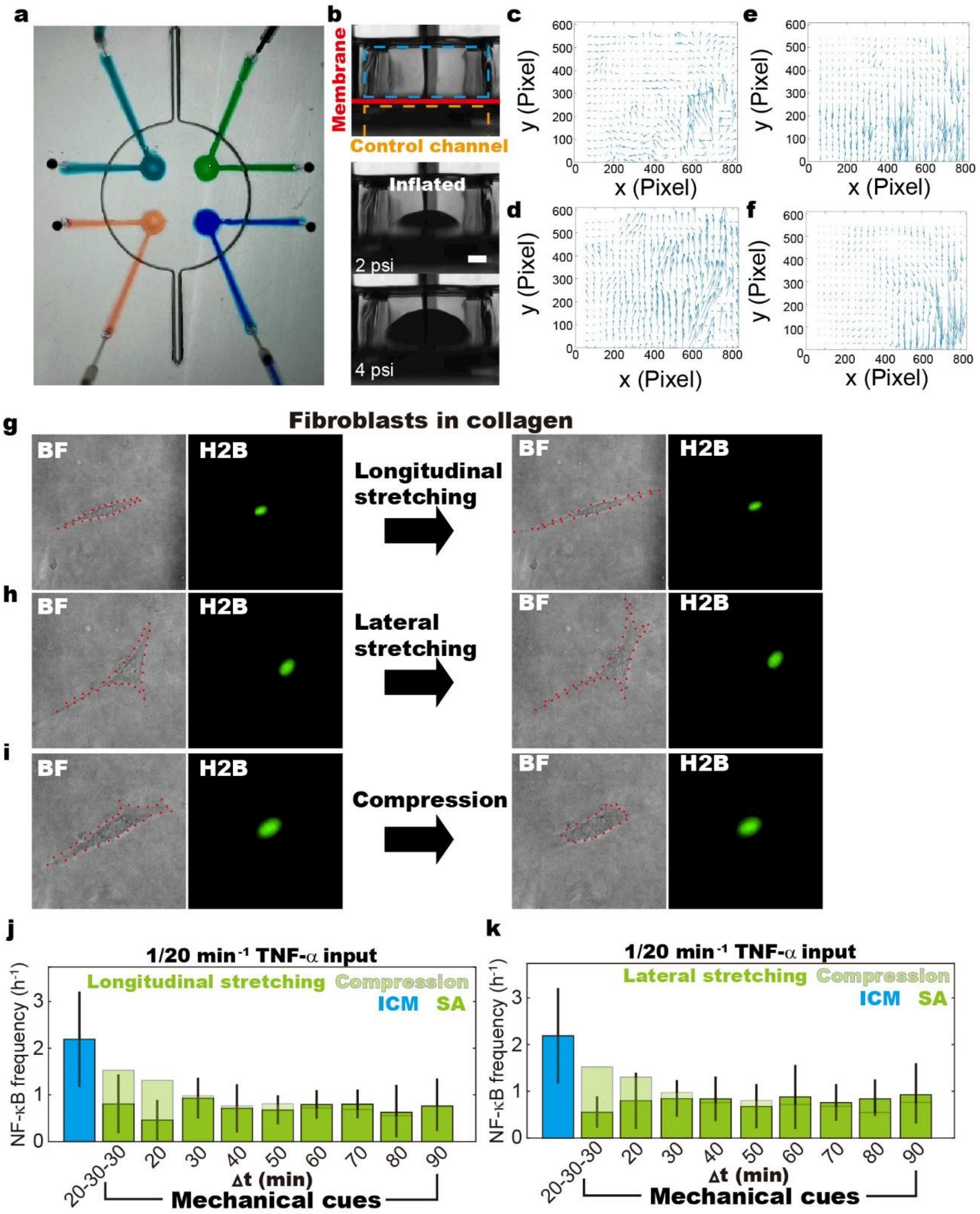

Figure S8. Morphological transitions of stand-alone (AS) cells induced by pressurizing the PDMS membrane, and consequently the remodeling of collagen fibers, Related to Figure 5a-b and d. a. The microfluidic chip is composed by a 4 stretchable PDMS membrane at the bottom of the culture chamber. b. Cross-sectional view of the fluidic chip with stretchable PDMS membrane. Fibroblasts are loaded, and maintained in the culture chamber (blue). PDMS membrane (red) is stretched by pressurizing the control channel (orange). Stretched PDMS membrane at pressure of 2 psi (left) and 4 psi (right). Scale bar denotes 200 μm. c-f. Distribution of the velocity field in the collagen matrix, which is generated by pressurizing different PDMS membranes, suggests various mechanical cues. g-i. Mechanical cues including pulling, stretching and compression are delivered to SA cells by inducing remodeling of collagen fibers.

j-k. By simultaneously introducing 1/20 min^{-1} TNF-α and different frequencies of collagen remodeling ranging from 1/20 to 1/90 min^{-1}, the effect of ICM contraction on NF-κB dynamics is systematically studied. It is revealed that when cells are either pulled or stretched, the induced mechanical cues bring no obvious changes as compared to the SA cells with no induced morphological transitions. Scale bar in Fig. b denotes 200 μm.

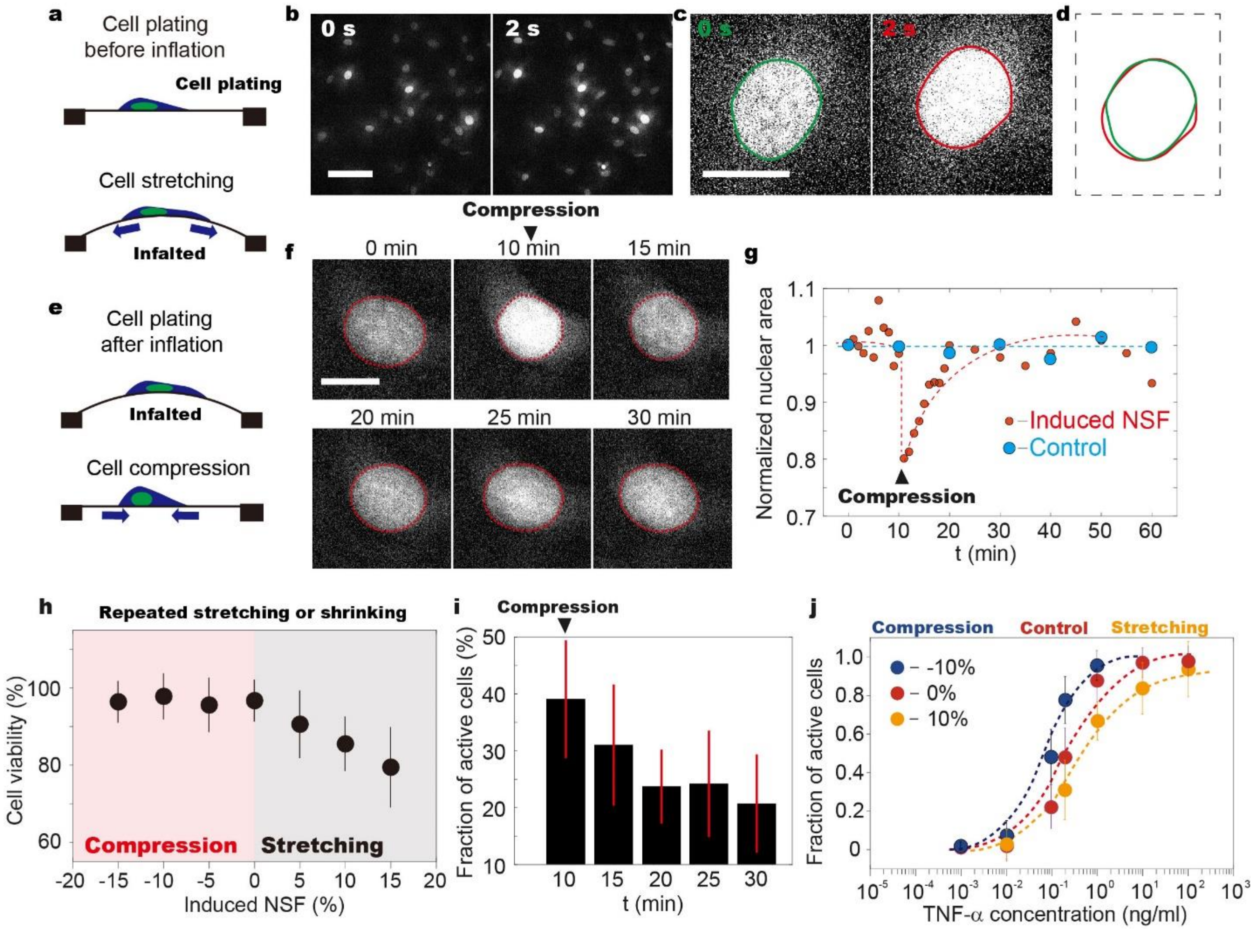

Figure S9. Responsiveness of SA cells with induced NSF, Related to Figure 6a. a. Schematic view showing that cell adhesion surface can be stretched when being plated on relaxed PDMS membrane, which is later inflated. b,c. Fluorescent images cells plating on a relaxed PDMS membrane, which is later inflated. d. Comparison between the outlines of individual cell at different timepoints shows distinctions in nuclear shape and area. e. To reach a relaxed state for the actin networks, cells were plated on an inflated PDMS membrane surface, which were then relaxed. f. Representative fluorescence images of nuclear shrinkage with decreased adhesion area. Cell gradually restore its original conformation within 20 min. g. Single cell traces showing nuclear shape variances with decreased (red) and unchanged (blue) adhesion area. All values were normalized by the initial nuclear area. The dashed lines were drawn for better visualization. h. Cell viability when being placed on repeatedly stretched or shrunk (at frequency of 1/20 min^{-1}) PDMS membrane for 2 hours. It is demonstrated that fibroblasts viability remains similar to control (~100%) when cell adhesion area repeatedly decreases. Cell viability decreases when being stretched, suggesting DNA damage induced by increased osmotic pressure at the top of chromatin. i. Responsiveness of fibroblasts to 0.1 ng/mL TNF-α stimulation at different stages of induced nuclear shape change. It is demonstrated that the increased responsiveness upon nuclear shape changes is only temporal. Fibroblasts restore the characteristics of isolated cells after 5 to 10 min. j. Fibroblasts are less responsive to TNF-α stimulation (single pulse) when nuclear area laterally expands by ~10%, and more when nuclear area decreases (~-10%). Scale bars denote 50 μm in Fig. b and 10 μm in Fig. c and Fig. f.
